# Comparative Safety of Pharmacologic Treatments for Persistent Depressive Disorder: A Systematic Review and Network Meta-Analysis

**DOI:** 10.1371/journal.pone.0153380

**Published:** 2016-05-17

**Authors:** Ramona Meister, Alessa von Wolff, Hannes Mohr, Martin Härter, Yvonne Nestoriuc, Lars Hölzel, Levente Kriston

**Affiliations:** 1 Department of Medical Psychology, University Medical Center Hamburg-Eppendorf, Hamburg, Germany; 2 Clinical Psychology and Psychotherapy, Institute of Psychology, University of Hamburg, Hamburg, Germany; 3 Department of Psychiatry and Psychotherapy, University Medical Center Freiburg, Freiburg, Germany; Western University of Health Sciences, UNITED STATES

## Abstract

We aimed to compare the safety of antidepressants for the treatment of persistent depressive disorder (PDD) with each other and with placebo. We conducted a systematic electronic search and included randomized controlled trials that investigated antidepressants for the treatment of PDD in adults. Outcomes were the incidence of experiencing any adverse event, specific adverse events and related treatment discontinuations. We analyzed the data using traditional and network meta-analyses. Thirty-four studies that comprised 4,769 patients and examined 20 individual agents in nine substance classes were included. Almost all analyzed substance classes were associated with higher discontinuation rates than placebo including tricyclic antidepressants (TCAs), selective serotonin reuptake inhibitors (SSRIs), monoamine oxidase inhibitors (MAOIs), antipsychotics, and the serotonin antagonist and reuptake inhibitor (SARI) trazodone. The odds of experiencing any adverse event were significantly higher for TCAs and serotonin noradrenaline reuptake inhibitors (SNRIs) compared to placebo. Pairwise comparisons among the substance classes revealed that more patients receiving TCAs or SNRIs experienced any adverse event and that more patients receiving TCAs or the SARI trazodone discontinued treatment. The complementary treatment with acetyl-l-carnitine showed lower rates of experiencing any adverse event and related discontinuations than all other comparators. TCAs were primarily associated with (anti-)cholinergic and sedating adverse events. SSRIs primarily showed gastrointestinal adverse events. Patients treated with the antipsychotic amisulpride were more likely to manifest weight gain and endocrine adverse events. The comparative evidence for further agents was insufficient or lacking. The identified safety differences may be used to inform the selection among the antidepressants.

## Introduction

During their lifetime, approximately 3% to 6% of the adults in Western countries suffer from a form of depression that persists for at least two years [1,2]. Within the literature, four subtypes of such persistent forms are distinguished: (a) a continuing mild depressive mood (dysthymia), (b) a state meeting all criteria for major depression continuously (chronic major depression), (c) a recurrent major depression with incomplete remission between episodes, and (d) a superimposition of a major depressive episode on an antecedent dysthymia (double depression) [2–4]. In the DSM-5, the new diagnostic category of persistent depressive disorder (PDD) was introduced subsuming those subtypes [4].

Systematic reviews, meta-analyses and clinical guidelines show evidence for the efficacy of pharmacological interventions in the treatment of PDD [5–11]. However, only few definite benefits of one antidepressive treatment over another could be determined [5,9–13]. A network meta-analysis based on the same set of primary studies like the present one revealed that among sufficiently tested agents the selective serotonin reuptake inhibitors (SSRI) fluoxetine, paroxetine, and sertraline, the monoamine oxidase inhibitor (MAO-I) moclobemide, the tricyclic antidepressant (TCA) imipramine, the serotonin receptor antagonist ritanserin, the antipsychotic amisulpride and the complementary treatment acetyl-l-carnitine were significantly more effective than placebo with hardly any differences between them [14]. When the evidence regarding efficacy does not warrant recommending a particular treatment, the issue of adverse events becomes more important as a basis for clinical decision-making [11,15–17].

In the treatment of major depressive disorder, differences have been found in the profiles of adverse events among substance classes. TCAs showed to have more sedating (e.g., somnolence), (anti-)cholinergic (e.g., dry mouth), and cardiovascular adverse events (e.g., palpitations). SSRIs in contrast were shown to have a higher occurrence of activating (e.g., insomnia) and gastrointestinal adverse events (e.g., nausea) [18–21]. Other substance classes such as MAOIs and serotonin noradrenaline reuptake inhibitors (SNRIs) appear to be as well-tolerated as SSRIs, although the evidence is still insufficient [22].

It remains unclear whether these findings may be transferred to the treatment of PDD. Because some differences exist in the efficacy of pharmacological interventions between acute and persistent forms of depression [3], it is possible that these conditions also differ regarding the adverse events experienced. Considering that expectations and conditioning processes contribute to the manifestation of adverse events [23–26], persistently depressed patients may be a particularly vulnerable patient group. Patients with PDD suffer from negatively biased cognitions and therefore negative expectations regarding treatment. They are likely to have received several unsuccessful prior treatments, during which they might still have experienced adverse events of the interventions (conditioning) [27,28]. At the same time, persistently depressive patients mostly require a long-term treatment, during which adverse events may still be persistent [29]. This poses a substantial burden. First, the depressive symptoms may worsen and quality of life may decrease because of burdensome adverse events [30]. Second, adverse events are the most common reason for non-adherence and discontinuation of antidepressive treatments [31,32].

Nevertheless, adverse events during pharmacologic interventions in the treatment of PDD have been insufficiently examined. Although the National Institute for Health and Clinical Excellence (NICE) highlights that SSRIs are better tolerated than TCAs by patients suffering from subthreshold depressive symptoms (including dysthymia) [12], they do not offer findings for other antidepressive substance classes, detailed information on specific adverse events, or a comparison of adverse events among individual agents. The present review aimed to update the existing comparative evidence on PDD by comparing SSRIs and TCAs regarding the number of patients experiencing any adverse event and discontinuing treatment due to adverse events and to enlarge it by considering all other available substance classes using network meta-analysis. Network meta-analysis allows the synthesis of information of both direct and indirect comparisons of interventions. Considering the large number of antidepressants and the relatively scant research in the field of PDD, this approach can deliver crucial information that could not have been obtained by an analysis of the direct evidence alone. Additionally, we aimed to investigate the comparative evidence concerning specific adverse events (e.g., headache) associated with individual agents using multiple traditional meta-analyses.

## Materials and Methods

We prepared the manuscript in accordance with the Preferred Reporting Items for Systematic Reviews and Meta-Analysis (PRISMA) statement incorporating network meta-analyses [33]. A study protocol was published apriori in an open-access journal [34].

### Eligibility criteria

We included randomized controlled trials that compared acute pharmacologic treatments for adults diagnosed with PDD with each other or with placebo. Reliance upon standardized criteria for the diagnosis was required. As the distinction between subtypes of persistent depressive disorder is controversial, inclusion was primarily driven by the duration of the existing depressive disorder of at least two years. Only studies that reported one of the following outcomes in each treatment arm were included: (a) the number of discontinuations due to adverse events, (b) the number of patients experiencing any adverse event, and (c) the number of patients experiencing specific adverse events.

### Search strategy

We systematically searched the following databases from inception through October 2014: Medline, Embase, PsycINFO, ISI Web of Science, the Cumulative Index to Nursing and AlliedHealth (CINAHL), BIOSIS, and the Cochrane Central Register of Controlled Trials (CENTRAL). We performed a primary search in 2010 and updates in 2013, 2014, and 2016. Additionally, we searched all volumes of the Archives of General Psychiatry, the Journal of Consulting and Clinical Psychology, and the Journal of Affective Disorders by hand, contacted the first author of each included study for more information regarding published and unpublished studies, and accomplished forward and backward citation tracking. See S1 File for the complete electronic data base search strategy.

### Study selection

One of two reviewers (RM, AvW) screened the titles and abstracts of all identified articles. Subsequently, two of six reviewers (LPH, HM, RM, LK, AvW, AW) independently examined the full texts of all the potentially relevant articles according to the predefined eligibility criteria. Disagreements were resolved by discussion until consensus was reached.

### Data collection and assessment of methodological quality

We extracted data on study characteristics including treatment characteristics, sample characteristics, and outcomes using a structured extraction form. We assessed the methodological quality of the included studies in accordance with the Cochrane Collaboration’s Risk of Bias tool that was modified and extended in accordance with the recommendations of the Cochrane Collaboration regarding adverse events [15] and the US Agency for Healthcare Research and Quality (AHRQ) [35] (see S1 Table for the modified and extended items). Two of five reviewers (RM, HM, LK, AvW, AW) conducted the data extraction and performed the assessment of methodological quality. Disagreements were resolved through discussions.

### Data synthesis and analysis

We summarized the outcomes using odds ratios (ORs) with corresponding 95% confidence intervals (CIs). We therefore calculated the odds of experiencing an adverse event in each treatment arm (e.g. the number of patients experiencing nausea in treatment arm A divided by the number of patients experiencing no nausea in treatment arm A) and divided the odds to obtain the odds ratio. For the numbers of patients experiencing specific adverse events and for those experiencing any adverse event, we calculated the odds ratios on the basis of the safety sample provided by the authors, respectively. We used the randomized sample size as the basis for the calculations of the odds of discontinuation due to adverse events. To address zero-cells, we applied a correction factor (0.5) [36,37].

For the number of patients experiencing any adverse event and the number of discontinuations due to adverse events, we conducted network meta-analyses using a graph-theoretical method by Rücker [38]. We calculated the odds of experiencing any adverse event and of treatment discontinuation due to adverse events for each substance class compared to placebo. Additionally, we conducted pairwise comparisons of all substance classes with each other. We examined heterogeneity and inconsistency by decomposing Cochran’s Q statistic [39,40].

For specific adverse events and head-to-head comparisons of individual agents, we conducted traditional meta-analyses if two or more studies provided the required data. In all analyses, we applied a random effects model with inverse variance weights [41]. For each meta-analysis, we estimated the extent of heterogeneity using the I^2^ statistic. All calculations were performed in the open source statistical environment R [42] with the packages netmeta [38,40] and metafor [43,44].

## Results

### Study selection and characteristics of included studies

**Fig 1:**
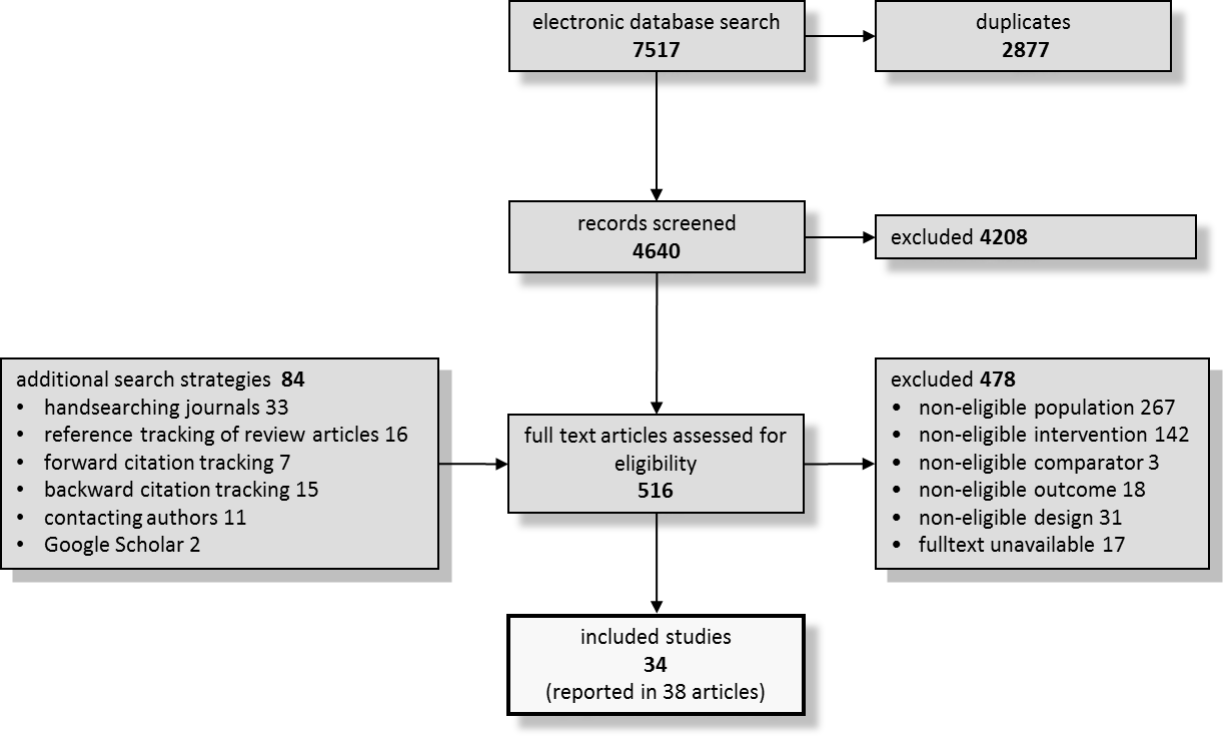
Study flow chart.

Altogether, we included 34 primary studies in the systematic review that comprised a total of 4,769 patients and examined 20 individual agents in nine substance classes. Fig 1 displays the study flow diagram that summarizes the results of the primary search in 2010 and the updates in 2013, 2014, and 2016 (more detailed flow diagrams are displayed in the S2 File). Nineteen and 28 studies provided data on the number of patients experiencing any adverse event and the number of discontinuations due to adverse events by treatment arm, respectively. Twenty-three of the included studies reported numerical data for specific adverse events.

The studies were published between 1987 and 2013. The sample sizes ranged from 30 to 635. The majority of the studies included dysthymic patients (24 of 34), five examined samples with dysthymic patients and patients suffering from double depression, one focused on patients with chronic major depression and the remaining four studies included dysthymic patients and patients with other forms of chronic depression. Most of the studies included only outpatients (28 of 34). The average age of the patients ranged from 29.5 to 75.0 years with a female predominance (37–94%). The reported dosages of antidepressants were in accordance with actual guidelines [9], and the treatment durations ranged from 4 to 24 weeks. Seventeen studies were conducted in European countries, whereas twelve were in North American countries and three were multicenter studies. Adverse events were frequently assessed using adverse events scales or checklists (6 of 34), unprompted patient reports (4 of 34), open questions (3 of 34) and combinations of these methods (7 of 34). However, in many of the studies the assessment methods were not clearly reported (12 of 34). A full reference list and detailed characteristics of the included studies are presented in the S3 File and the S2 Table, respectively.

Global methodological quality was rated as *low* for 13 studies, as *unclear* for 17 studies and as *high* for 4 studies. Few studies reported an adequate generation of the allocation sequence (8 of 34) and only one study an adequate concealment of allocation. Most studies did not exclude patients from the adverse event analysis or addressed their exclusion adequately (24 of 34). Twenty-nine studies reported an adequate blinding of patients and clinicians; however, only 2 studies reported an adequate blinding of the adverse events assessor. More than half of the studies defined the sample of adverse events they reported (e.g., adverse events reported by at least 2% of the patients; 21 of 34), but only eight defined the specific adverse events themselves. Fifteen of the studies used adequate methods for monitoring adverse events. The results of the assessment of methodological quality for the individual studies are presented in the S3 Table.

### Treatment discontinuation due to adverse events and experiencing any adverse event for substance classes

**Fig 2:**
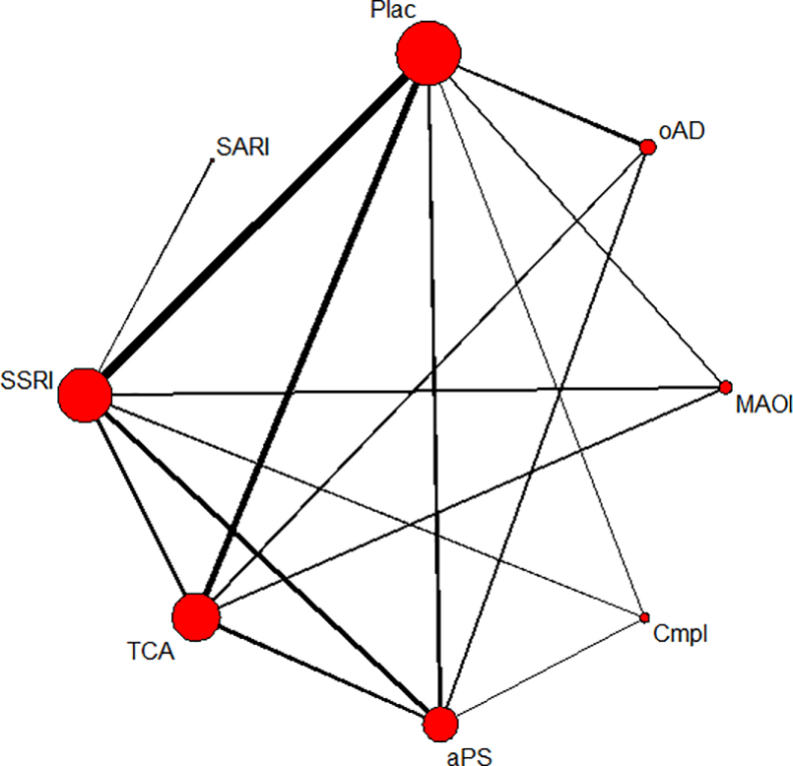
Network of eligible comparisons on discontinuations due to adverse events. The line width is proportional to the number of studies that compare each pair of treatments, and the size of each node is proportional to the number of comparisons the treatment is included in. Plac = placebo; oAD = other antidepressant; MAOI = monoamine oxidase inhibitor; Cmpl = complementary treatment; aPS = antipsychotic; TCA = tricyclic antidepressant; SSRI = selective serotonin reuptake inhibitor; SARI = serotonin antagonist reuptake inhibitor.

**Fig 3:**
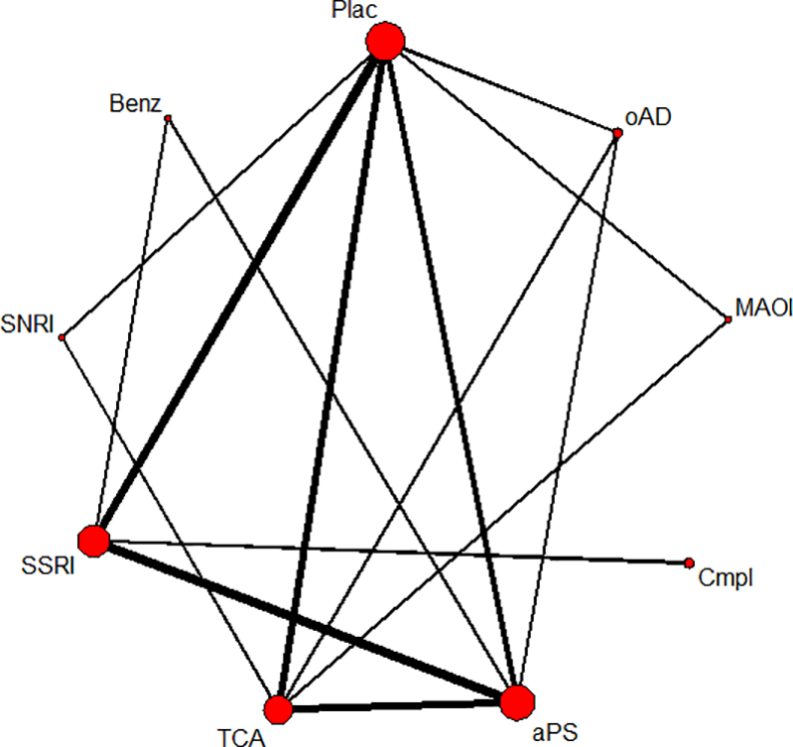
Network of eligible comparisons on experiencing any adverse event. The line width is proportional to the number of studies that compare each pair of treatments, and the size of each node is proportional to the number of comparisons the treatment is included in. Plac = placebo; oAD = other antidepressant; MAOI = monoamine oxidase inhibitor; Cmpl = complementary treatment; aPS = antipsychotic; TCA = tricyclic antidepressant; SSRI = selective serotonin reuptake inhibitor; SNRI = serotonin noradrenalin reuptake inhibitor; Benz = benzodiazepine.

Twenty-eight studies including 4,276 patients in 61 treatment arms contributed to the network meta-analysis of discontinuation rates due to adverse events. In the network of patients experiencing any adverse event, 19 studies with 2,944 patients in 42 treatment arms were included (see Figs 2 and 3 for network graphs). Examined substance classes were SSRIs (18 studies on sertraline, fluoxetine, escitalopram and paroxetine), TCAs (eleven studies on imipramine, amineptine and amitriptyline), antipsychotics (nine studies on amisulpride and flupenthixol), MAOIs (three studies on moclobemide and phenelzine), SNRIs (two studies on duloxetine and reboxetine) and the complementary treatment acetyl-l-carnitine (three studies). The serotonin antagonist and reuptake inhibitor (SARI) trazodone and the benzodiazepine lorazepam were investigated in one study, respectively; four additional studies investigated other antidepressants, namely ritanserin and minaprine. Placebo was used as a comparator in 17 studies.

**Fig 4:**
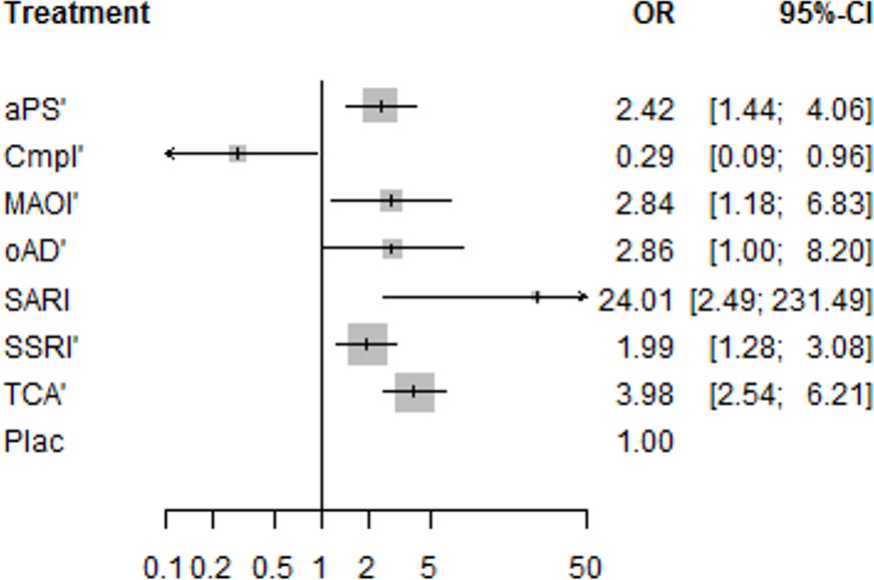
Network meta-analysis estimates of discontinuation rates due to adverse events for substance classes compared with placebo. Odds ratios higher than 1 reflect a higher discontinuation rate due to adverse events in the substance class arms, whereas odds ratios lower than 1 reflect a higher discontinuation rate due to adverse events in the placebo arms; OR = odds ratio; CI = confidence interval; aPS = antipsychotics (containing amisulpride and flupenthixol); Cmpl = complementary treatments (containing acetyl-l-carnitine); MAOI = monoamine oxidase inhibitors (containing moclobemide and phenelzine); oAD = other antidepressants (containing ritanserin and minaprine); Plac = placebo, sari = serotonin antagonist and reuptake inhibitor (containing trazodone); SSRI = selective serotonin reuptake inhibitors (containing sertraline, fluoxetine, escitalopram, and paroxetine); TCA = tricyclic antidepressants (containing imipramine, amineptine, and amitriptyline); ‘contrasts that are informed by at least one direct comparison to placebo.

Patients treated with almost all substance classes *discontinued treatment due to adverse events* significantly more often than patients treated with placebo, including antipsychotics (OR = 2.42), MAOIs (OR = 2.84), SSRIs (OR = 1.99), TCAs (OR = 3.98) and the SARI trazodone (OR = 24.01). The complementary treatment acetyl-l-carnitine showed a significantly lower rate of discontinuations due to adverse events than placebo. For the other antidepressants ritanserin and minaprine together no significant differences compared to placebo could be detected (Fig 4).

In pairwise comparisons, TCAs showed a higher discontinuation rate than SSRIs (OR = 2.00), antipsychotics (OR = 1.64) and the complementary treatment acetyl-l-carnitine (OR = 13.67). Furthermore, the SARI trazodone led to more discontinuations than SSRIs (OR = 12.5), antipsychotics (OR = 9.93), and the complementary treatment acetyl-l-carnitine (OR = 82.58). Pairwise comparisons also revealed that MAOIs (OR = 9.77), antipsychotics (OR = 8.33), SSRIs (OR = 6.83) and other antidepressants (OR = 10.00) were significantly more often associated with treatment discontinuations due to adverse events than the complementary treatment acetyl-l-carnitine (Table 1). In this analysis, we did not find any evidence for heterogeneity (Q = 5.42, df = 11, p = 0.91) or inconsistency (Q = 5.54, df = 15, p = 0.99).

**Table 1:** Pairwise comparison between substance classes regarding discontinuation rates due to adverse events (below the diagonal) and rates of patients experiencing at least one adverse event (above the diagonal).

**TCA**	**0.51[0.32–0.80]**	1.24’[0.57–2.68]	-	0.53’[0.27–1.03]	0.36[0.08–1.63]	**0.12[0.06–0.27]**	**0.50’[0.34–0.73]**	0.64’[0.32–1.30]
**2.00’[1.40–2.87]**	**SSRI**	2.44[1.02–5.85]	-	1.05[0.51–2.15]	0.70’[0.16–3.07]	**0.25’[0.12–0.51]**	0.98’[0.70–1.38]	1.27[0.60–2.70]
-	**-**	**SNRI**	**-**	0.43[0.16–1.17]	0.29[0.05–1.57]	**0.10[0.03–0.30]**	**0.40[0.17–0.94]**	0.52[0.19–1.47]
0.17[0.02–1.57]	**0.08’[0.01–0.76]**	**-**	**SARI**^**+**^	-	-	-	-	-
1.40’[0.64–3.08]	0.70’[0.30–1.64]	-	8.44[0.78–91.29]	**MAO-I**^**+**^	0.67[0.13–3.41]	**0.24[0.09–0.63]**	0.94[0.47–1.90]	1.21[0.48–3.09]
**-**	**-**	**-**	**-**	**-**	**Benz**^**+**^	0.25[0.07–1.77]	1.40’[0.32–6.14]	1.81[0.35–9.22]
**13.67[4.29–43.55]**	**6.83’[2.16–21.60]**	**-**	**82.58[6.75-10e]**	**9.77[2.42–39.44]**	**-**	**Cmpl**^**+**^	**4.00’[1.98–8.10]**	**5.16[1.92–13.86]**
**1.64’[1.10–2.45]**	0.82’[0.55–1.23]	**-**	**9.93[1.03–95.10]**	1.18[0.49–2.82]	**-**	**0.12’[0.04–0.36]**	**aPS**	1.29’[0.64–2.60]
1.39’[0.51–3.76]	0.69[0.25–1.95]	-	8.40[0.72–97.49]	0.99[0.28–3.52]	**-**	**0.10[0.02–0.46]**	0.85’[0.30–2.37]	**oAD**

Odds ratios with 95% confidence intervals are displayed; estimates refer to a comparison of the column-defined substance class with the row-defined substance class and are obtained from network meta-analysis; odds ratios higher than 1 refer to a higher rate of discontinuation due to adverse events and a higher rate of patients with at least one adverse event in the column-defining substance class, respectively; odds ratios lower than 1 refer to a higher rate of discontinuation due to adverse events and a higher rate of patients with at least one adverse event in the row-defining substance class, respectively; statistically significant estimates are boldfaced; ‘contrasts that are informed by at least one direct comparison; ^+^substance classes that contain only one agent in either analysis.

TCA = tricyclic antidepressants (containing imipramine, amineptine, and amitriptyline); SSRI = selective serotonin reuptake inhibitors (containing sertraline, fluoxetine, escitalopram, and paroxetine); SNRI = serotonin noradrenaline reuptake inhibitor (containing reboxetine and duloxetine); SARI = serotonin antagonist and reuptake inhibitor (containing trazodone); MAOI = monoamine oxidase inhibitors (containing moclobemide and phenelzine); Benz = benzodiazepine (containing lorazepam); Cmpl = complementary treatments (containing acetyl-l-carnitine); aPS = antipsychotics (containing amisulpride and flupenthixol); oAD = other antidepressants (containing ritanserin and minaprine.

**Fig 5:**
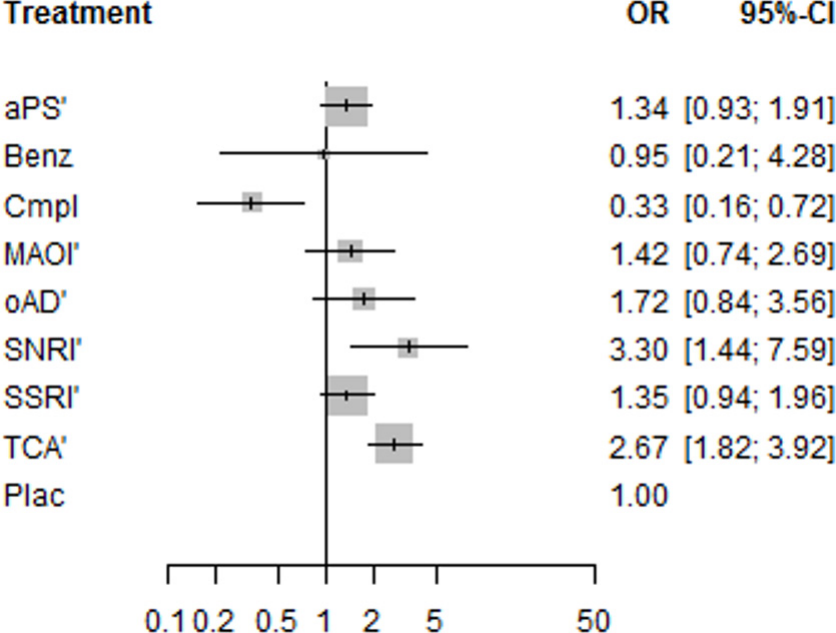
Network meta-analysis estimates of experiencing any adverse event for substance classes compared with placebo. Odds ratios higher than 1 reflect a higher rate of patients experiencing at least one adverse event in the substance class arms, and odds ratios lower than 1 reflect a higher rate of patients experiencing at least one adverse event in the placebo arms; OR = odds ratio; CI = confidence interval; aPS = antipsychotics (containing amisulpride and flupenthixol); Benz = benzodiazepine (containing lorazepam); Cmpl = complementary treatments (containing acetyl-l-carnitine); MAOI = monoamine oxidase inhibitors (containing moclobemide); oAD = other antidepressants (containing ritanserin and minaprine); Plac = placebo; SNRI = serotonin noradrenalin reuptake inhibitor (containing duloxetine and reboxetine); SSRI = selective serotonin reuptake inhibitors (containing sertraline, fluoxetine, escitalopram, and paroxetine); TCA = tricyclic antidepressants (containing imipramine, amineptine, and amitriptyline); ‘contrasts that are informed by at least one direct comparison to placebo.

Network meta-analysis showed that patients treated with SNRIs (OR = 3.30) or TCAs (OR = 2.67) more often *experienced any adverse event* than patients treated with placebo. Patients treated with the complementary treatment acetyl-l-carnitine less often reported any adverse event than patients treated with placebo (OR = 0.33). For antipsychotics, SSRIs, the benzodiazepine lorazepam, the MAOI moclobemide and other antidepressants no differences compared to placebo were observed (Fig 5).

In pairwise comparisons, TCAs were observed to have a significantly higher rate of patients experiencing any adverse event than SSRIs (OR = 1.96), antipsychotics (OR = 2.00), and the complementary treatment acetyl-l-carnitine (OR = 8.33). Pairwise comparisons revealed that SSRIs (OR = 4.00), SNRIs (OR = 10.00), MAOIs (OR = 4.17), antipsychotics (OR = 4.00), and other antidepressants (OR = 5.16) were associated with a higher rate of patients experiencing any adverse event than the complementary treatment acetyl-l-carnitine. Furthermore, SNRIs showed a significantly higher adverse event rate than antipsychotics (OR = 2.5) (Table 1). In this network, we did not find evidence for inconsistency (Q = 3.82, df = 8, p = 0.87); however, we identified some heterogeneity (Q = 15.93, df = 7, p = 0.03). A detailed investigation revealed that this could be traced back to two conflicting three-arm studies that compared TCAs, antipsychotics, and placebo [45–47]. For the comparison of antipsychotics with placebo, one study tended to favor antipsychotics and the other placebo, whereas for the other two comparisons (TCAs vs. antipsychotics and placebo, respectively) the effect estimates varied in their extent, though not in their direction.

### Specific adverse events for individual agents

Twenty-three studies including 3,840 patients in fifty treatment arms reported on 77 specific adverse events. Examined agents were the TCA imipramine (six studies), the antipsychotic amisulpride (seven studies), the SSRIs fluoxetine (seven trials) and sertraline (five trials), the MAOI moclobemide (two studies), and the complementary treatment acetyl-l-carnitine (two studies). One study investigated amitriptyline, clomipramine, flupenthixol, duloxetine, reboxetine, viloxazine, paroxetine, escitalopram, minaprine, lorazepam, and ritanserin, respectively. Nine studies compared one individual agent with placebo. In total, the studies comprised 315 head-to-head comparisons allowing for 39 meta-analyses (i.e., at least two studies for a comparison).

**Fig 6:**
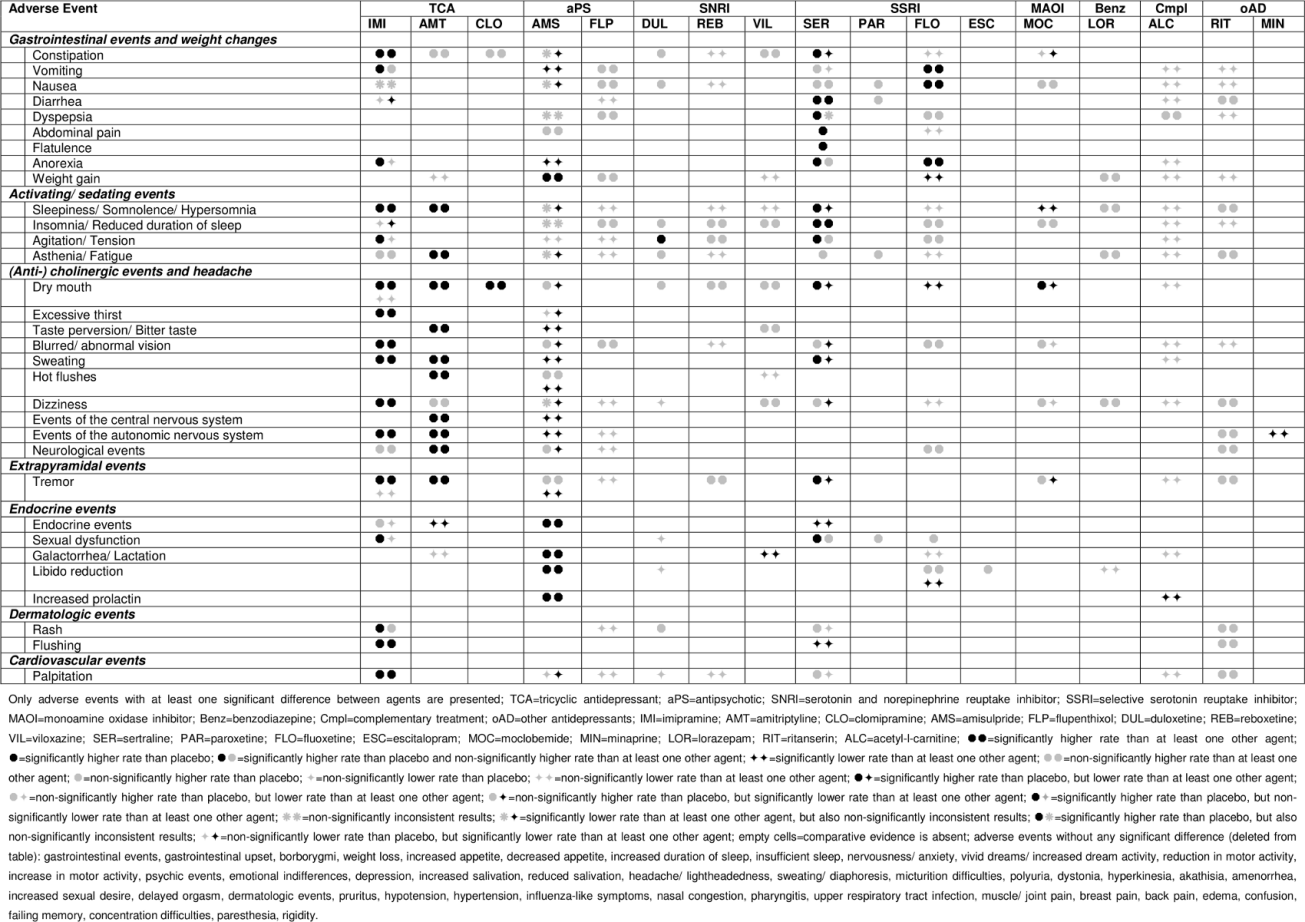
Adverse event profiles for all investigated agents.

Fig 6 presents the adverse event profiles of all investigated agents. Only adverse events with at least one significant difference between agents are presented (see S4 Table for the adverse event profiles with all reported adverse events). For several agents and adverse events, comparative evidence is completely lacking (empty cells in Fig 6) or is insufficient to draw firm conclusions (grey symbols in Fig 6; CIs include 1). Thus, we only report on differences that reached statistical significance (black symbols in Fig 6) and present the results qualitatively (see S5 Table for the ORs with corresponding 95% CIs of all head-to-head comparisons). Heterogeneity was low in 35 (I^2^ = 0 to 40.8%) and substantial in four (I^2^ = 63.5 to 81.9%) of the conducted meta-analyses. Substantial heterogeneity was present in two meta-analyses of imipramine compared with placebo (constipation and tremor) and two meta-analyses of sertraline compared with placebo (sweating and dizziness).

The findings suggest that the *TCAs* imipramine and amitriptyline have comparable adverse event profiles, whereas evidence for clomipramine is largely absent. Both imipramine and amitriptyline showed mainly sedating (sleepiness and fatigue) and (anti-)cholinergic (dry mouth, excessive thirst, bitter taste, blurred vision, sweating, hot flushes, and dizziness) adverse events in comparison to other agents. Some extrapyramidal (tremor), dermatologic (rash and flushing), gastrointestinal (constipation), and cardiovascular (palpitation) adverse events also occurred more frequently in treatments with imipramine or amitriptyline than in other agents.

The *antipsychotic* amisulpride predominantly produced endocrine events (galactorrhea, libido reduction, increased prolactin) and weight gain compared to other agents. Regarding other adverse events, amisulpride was shown to be favorable. For flupenthixol, the randomized evidence was insufficient.

The adverse event profiles of the *SSRIs* sertraline and fluoxetine were similar; for paroxetine and escitalopram, however, comparative evidence is largely absent. Primarily, sertraline and fluoxetine were associated with more gastrointestinal (nausea, vomiting, diarrhea, anorexia) and activating (insomnia and agitation) adverse events compared to placebo and other agents. For sertraline, some (anti-)cholinergic, extrapyramidal and endocrine adverse events were observed more often than for placebo.

The *SNRI* duloxetine appears to be associated with more agitation than placebo and the *MAOI* moclobemide with more experiences of dry mouth than placebo, though less than other agents. Further comparative evidence for duloxetine and moclobemide as well as for *other treatments that were investigated in at least one included study*, such as the SNRIs reboxetine and viloxazine, the benzodiazepine lorazepam, the complementary treatment acetyl-l-carnitine and the other antidepressants minaprine and ritanserin is either absent or insufficient.

## Discussion

### Summary of findings

In the present study, we investigated the adverse event profiles of antidepressive substance classes and individual agents and identified some differences among them. The findings suggest that almost all substance classes led to higher discontinuation rates due to adverse events than placebo including TCAs, SSRIs, MAOIs, antipsychotics and the SARI trazodone. Conversely, other antidepressants (acetyl-l-carnitine, minaprine, and ritanserin) did not show increased discontinuation rates. The odds of experiencing any adverse event were higher for TCAs and SNRIs and lower for acetyl-l-carnitine than for placebo. Other substance classes showed no difference compared to placebo. Thus, even though the odds of experiencing at least one adverse event were comparable to placebo in most of the substance classes, adverse events in some substance classes seem to be more burdensome making a treatment discontinuation more likely. Pairwise comparison of substance classes suggested that TCAs and the SARI trazodone are associated with the highest and the complementary treatment acetyl-l-carnitine with the lowest discontinuation rates due to adverse events. The odds of developing any adverse event were highest for TCAs and SNRIs and lowest for acetyl-l-carnitine compared to other substance classes.

We could show that differences between individual agents belonging to the same class were largely negligible. Whereas patients treated with the TCAs imipramine and amitriptyline primarily manifested (anti-)cholinergic, sedating and dermatologic adverse events together with constipation, palpitation and tremor, the SSRIs sertraline and fluoxetine were primarily associated with gastrointestinal adverse events. Regarding multiple adverse events, the use of the antipsychotic amisulpride was shown to be favorable, though likely to be associated with weight gain and endocrine adverse events. Unfortunately, comparative evidence for additionally investigated agents was insufficient or completely lacking to draw firm conclusions and to describe the pattern of adverse events. These findings are in accord with results on the comparative safety of antidepressants in treating acute forms of depression [18,20–22,29].

### Limitations

In the present study, we investigated the comparative safety of pharmacological treatments for PDD by meta-analyzing randomized controlled trials. PDD was introduced as a new diagnostic category in the DSM-5. Subsuming dysthymic as well as chronic major depressive disorders, the diagnosis of PDD is heterogeneous and its validity has therefore been challenged [48]. A comparison of subtypes could have been helpful in validating the PDD approach. The numbers of included studies, however, were too small to allow sub-analyses. Consequently, this limitation needs to be considered when interpreting the results.

Another limitation to our findings is the insufficient comparative evidence from the *primary studies* for many substance classes, individual agents and (specific) adverse events. First, the number of included studies reporting on adverse events and thus the number of direct comparisons was relatively small. In the included studies, agents largely belonging to the substance classes TCAs, SSRIs and antipsychotics were compared with each other or with placebo. Agents belonging to other substance classes (e.g., SNRI, SARI, MAO-I), however, were primarily compared to placebo; thus head-to-head comparisons are missing. Additionally, for some substance classes (complementary treatment, SARI, benzodiazepine, and MAOI) only one agent could be considered in some or all analyses (acetyl-l-carnitine, trazodone, lorazepam, and moclobemide, respectively). Although, we found the differences between agents respectively belonging to the substance classes TCAs and SSRIs to be negligible, conclusions about the other substance classes might be problematic and may only be valid for the analyzed agents. It is further notable that for some treatments we could not identify any eligible randomized controlled trials. The consequence is that many commonly used treatment options such as the noradrenergic and specific serotonergic antidepressant (NaSSA) mirtazapine, the norepinephrine-dopamine reuptake inhibitor (NDRI) bupropion or the SSRI citalopram could not be investigated in our analyses. On the other hand, some of the included agents like the SNRI viloxazine is not approved for marketing anymore. A second flaw of the primary studies is that almost all studies were efficacy studies and were insufficiently powered to detect differences regarding adverse events. Although we aimed to increase power by pooling the data (if possible), some differences might have remained undetected. Third, the methodological quality was rated as low or unclear for the majority of the primary studies, including varying strategies to assess adverse events. Although the quality ratings may partially be explained by insufficient reporting practices (and not methodological quality) of the primary studies [49], our findings are limited. These limitations contribute to the fact that the 95% confidence intervals of various estimates were broad and the results must be judged as *imprecise*. When interpreting the results, it should therefore be considered that absent or insufficient comparative evidence regarding adverse events is not equivalent to evidence of the absence of differences among substance classes or individual agents.

Moreover, most primary studies reported on the frequency, though not on the severity or the time course of adverse events, which largely influences the burden of adverse events. For example, the burden might be minor, when adverse events occur temporarily (e.g., nausea, vomiting, agitation) and larger, when they occur continuously (e.g., sexual problems). This is particularly relevant for patients suffering from persistent forms of depression and requiring long-term treatments. This limitation may be additionally attributed to the fact that we only included randomized controlled trials investigating the acute treatment of PDD.

In the network meta-analyses, we did not detect statistically significant *inconsistency*. As the power to detect important inconsistency is frequently low, the results should not be interpreted as clear evidence of consistency. In the network of comparisons of experiencing any adverse event and in four comparisons of individual agents and specific adverse events, however, some *heterogeneity* was present. Heterogeneity in the network could be traced back to two studies comparing TCAs, antipsychotics and placebo. For the comparisons TCAs vs. antipsychotics and TCAs vs. placebo, the estimates varied to some extent, but were consistent in their direction and significance. Thus, confidence in the general conclusion regarding these comparisons is not reduced. For the comparison antipsychotics vs. placebo, however, the direction of the estimates was contradicting, reducing the confidence in the pooled estimate substantially. The reduced confidence is reflected in the broad 95% confidence interval including one. Comparing individual agents and specific adverse events, evidence that imipramine shows a higher rate of constipation and tremor and that sertraline shows higher rates of sweating and dizziness than placebo need to be interpreted with caution due to moderate to high heterogeneity.

Our findings are additionally limited by the fact that we did not search for *unpublished studies* extensively, which might have biased our results (publication bias). However, due to the strict inclusion criteria, all of the primary studies were sufficiently similar with respect to population, intervention and outcome so that no concerns about *indirectness* or *intransitivity* emerged.

In the present study, we examined the comparative safety of pharmacologic treatments for PDD. Beside pharmacologic treatments, current clinical practice guidelines recommend the use of psychotherapeutic and combined treatments for PDD [9,10,12]. For those treatments the number of patients with an adverse event and associated discontinuations are of high importance, too. With concurrent use of psychotherapy, for example, discontinuation of pharmacologic treatments may be less frequent [50]. However, primary studies on adverse events during psychotherapeutic and combined treatments for PDD are rare and no definite conclusions can be drawn [49].

### Conclusions

TCAs were shown to have a less favorable adverse event profile than other substance classes and should only be prescribed for well-justified reasons. The complementary treatment acetyl-l-carnitine was effective in previous studies [11] and was well tolerated in the present study. It might therefore be considered as a promising option in the treatment of PDD. However, further research is needed to confirm our results. Unfortunately, comparative evidence regarding specific adverse events for acetyl-l-carnitine is largely insufficient and cannot be used to inform clinical decision-making.

For the SSRIs sertraline and fluoxetine, the TCAs imipramine and amitriptyline, and the antipsychotic amisulpride, however, the pattern of specific adverse events can serve as a basis for clinical decision-making. The activating profile of both SSRIs might negatively influence the rhythm of sleep and wakefulness and might contribute to a worsening of the depressive symptoms, particularly for depressive patients suffering from insomnia and agitation. On the other hand, patients with reduced motivation may benefit from this adverse event profile. The gastrointestinal adverse events and notably the weight loss of both sertraline and fluoxetine might influence the depressive symptoms both negatively and positively, as well. Patients suffering from atypical depression often experience weight gain and may consider such adverse events as favorable. Although the TCAs imipramine and amitriptyline cause multiple burdensome adverse events and may therefore be less suitable as a treatment of PDD, the sedating adverse events may be beneficial and relieving for persistently depressed patients suffering from insomnia and agitation. Moreover, the antipsychotic amisulpride was found to be favorable regarding multiple adverse events, though it showed a high rate of endocrine adverse events including galactorrhea and libido reduction. Endocrine symptoms specifically are supposed to be particularly burdensome, which makes antipsychotics less recommendable [31]. Benzodiazepines were observed to be associated with few specific adverse events in our analyses. However, those agents should only be prescribed in exceptional cases due to their high risk of dependency [51].

Our safety findings are comparable to those for major depressive disorder and imply that safety findings for major depressive disorder are likely to be transferable to PDD. This might be particularly useful for treatment comparisons that were not sufficiently investigated for PDD. However, we investigated the comparative safety of pharmacological treatments. Differences in the absolute frequency of adverse events between acute and persistent depressive disorder might still exist (e.g. adverse event rates are generally higher or lower for PDD compared to major depressive disorder).

We additionally revealed some evidence gaps for the treatment of PDD. To enable direct comparisons of agents, further studies should focus on head-to-head comparisons, particularly of agents other than those belonging to SSRIs, TCAs and antipsychotics. In such studies, the standards of both assessing and reporting adverse events need to be improved [49]. Studies need to assess adverse events using a standardized checklist in a double-blind process. The reporting on adverse events should, additionally, follow actual guidelines such as the extension of the CONSORT statement regarding the reporting of harms, including the rates of patients experiencing specific and total adverse events per treatment [52].

In our systematic review on primary studies investigating the comparative safety of acute treatments of PDD, we demonstrated that substantial differences between both substance classes and individual agents exist. This information may be used to achieve the best possible fit between the effects (both positive and negative) of the agent and the individual needs of the patient.

## Supporting Information

S1 PRISMA Checklist PRISMA Checklist. (DOC)

S1 File Electronic database search strategy. (DOCX)

S2 File Study flow diagram. (DOCX)

S3 File Reference list. (DOCX)

S1 Table Assess methodological quality. (DOCX)

S2 Table Study characteristics and data. (DOCX)

S3 Table Methodological quality. (DOCX)

S4 Table Complete adverse event profiles. (DOCX)

S5 Table Odds ratios for all comparisons. (DOCX)

S6 Table AE data. (DOCX)
