# Supplementary material for: Comparative Safety of Pharmacologic Treatments for Persistent Depressive Disorder: A Systematic Review and Network Meta-Analysis
Source: PLoS One. 2016 May 17;11(5):e0153380. doi: 10.1371/journal.pone.0153380 (PMC4871495; doi:10.1371/journal.pone.0153380)
Supplement: S1 File — (DOCX) [file pone.0153380.s002.docx]

# S1 File. Electronic database search strategy

***MEDLINE***

Date of search: 18.01.2010, updated:18.01.2013, updated: 24.10.2014, updated: 02.03.2016

(((chron$ adj3 depress$) or dysthym$ or (double adj1 depress$) or (treatment adj1 resist$ adj1 depress$) or (non adj1 respon$ adj3 depress$) or (recurrent adj3 depress$)).ab,ti,sh.) AND ((random$ or rct).ab,ti. or random$.sh.)

***EMBASE***

Date of search: 18.01.2010, updated:18.01.2013, updated: 24.10.2014

(((chron$ adj3 depress$) or dysthym$ or (double adj1 depress$) or (treatment adj1 resist$ adj1 depress$) or (non adj1 respon$ adj3 depress$) or (recurrent adj3 depress$)).ab,ti,sh.) AND ((random$ or rct).ab,ti. or random$.sh.)

***PsycInfo***

Date of search: 18.01.2010, updated:18.01.2013, updated: 24.10.2014

(((chron$ adj3 depress$) or dysthym$ or (double adj1 depress$) or (treatment adj1 resist$ adj1 depress$) or (non adj1 respon$ adj3 depress$) or (recurrent adj3 depress$)).ab,ti,sh.) AND ((random$ or rct).ab,ti. or random$.sh.)

***ISI Web of Science***

Date of search: 19.01.2010, updated: 18.01.2013

(TS=(("chron* depress*") or dysthym* or ("double depress*") or ("treatment resist* depress*") or ("non respon* depress*") or ("recurrent depress*")) OR TI=(("chron* depress*") or dysthym* or ("double depress*") or ("treatment resist* depress*") or ("non respon* depress*") or ("recurrent depress*")) ) AND (TS=(random$ or rct) OR TI=(random$ or rct)))

Modified search strategy for update: 24.10.2014

(TS=(("chron* depress*") or dysthym* or ("double depress*") or ("treatment resist* depress*") or ("non respon* depress*") or ("recurrent depress*")) OR TI=(("chron* depress*") or dysthym* or ("double depress*") or ("treatment resist* depress*") or ("non respon* depress*") or ("recurrent depress*")) ) AND (TS=(random* or rct) OR TI=(random* or rct))

***CINAHL***

Date of search: 18.01.2010, updated: 18.01.2013, updated: 24.10.2014

((TI “chron* depress*” or TI dysthym* or TI “double depress*” or TI “treatment resist* depress*” or TI “non respon* depress*” or TI “recurrent depress*”) OR (AB “chron* depress*” or AB dysthym* or AB “double depress*” or AB “treatment resist* depress*” or AB “non respon* depress*” or AB “recurrent depress*” ) OR (MW “chron* depress*” or MW dysthym* or MW “double depress*” or MW “treatment resist* depress*” or MW “non respon* depress*” or MW “recurrent depress*” )) AND (TI rct or AB rct or MW rct or TI random* or AB random* or MW random* )

***BIOSIS***

up to 2004 via OVID

Date of search: 18.01.2010, updated: 18.01.2013

((chron$ adj3 depress$) or dysthym$ or (double adj1 depress$) or (treatment adj1 resist$ adj1 depress$) or (non adj1 respon$ adj3 depress$) or (recurrent adj3 depress$)).ab,ti,sh. and ((random$ or rct).ab,ti. or random$.sh.) 5

from 2005 via DIMDI

Date of search: 25.01.2010, updated: 18.01.2013, updated: 24.10.2014

((FT=random* OR FT=rct ) AND PY=2005 to 2010 AND (LA=ENGLISH OR LA=GERMAN) AND pps=Mensch) AND ((((((FT="chron* depress*" OR FT=dysthym*) OR FT="double depress*" ) OR FT="treatment resist* depress*" ) OR FT="non respon* depress*" ) OR FT="recurrent depress*" ) AND PY=2005 to 2010 AND (LA=ENGLISH OR LA=GERMAN) AND pps=Mensch )

***CENTRAL***

Date of search: 19.01.2010, updated: 18.01.2013, updated: 24.10.2014

((“chron* depress*”):ti,ab,kw or (dysthym*):ti,ab,kw or (“double depress*”):ti,ab,kw or (“treatment resist* depress*”):ti,ab,kw or (“non respon* depress*”):ti,ab,kw (“recurrent depress*”):ti,ab,kw in Clinical Trials) AND ((random*):ti,ab,kw or (rct):ti,ab,kw in Clinical Trial
