# Supplementary material for: Comparative Safety of Pharmacologic Treatments for Persistent Depressive Disorder: A Systematic Review and Network Meta-Analysis
Source: PLoS One. 2016 May 17;11(5):e0153380. doi: 10.1371/journal.pone.0153380 (PMC4871495; doi:10.1371/journal.pone.0153380)

# S2 Figure. Study flow diagram

***A: Primary search (2010)***


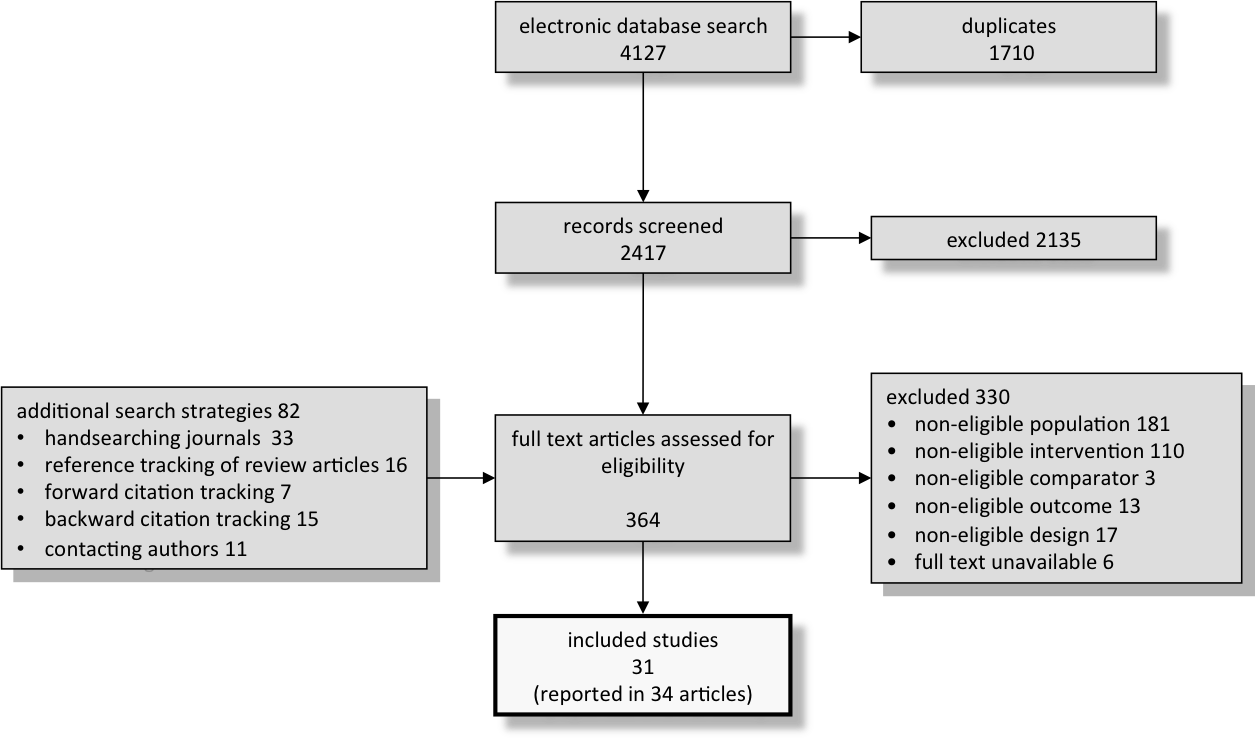


***B: Search update (2013)***

***
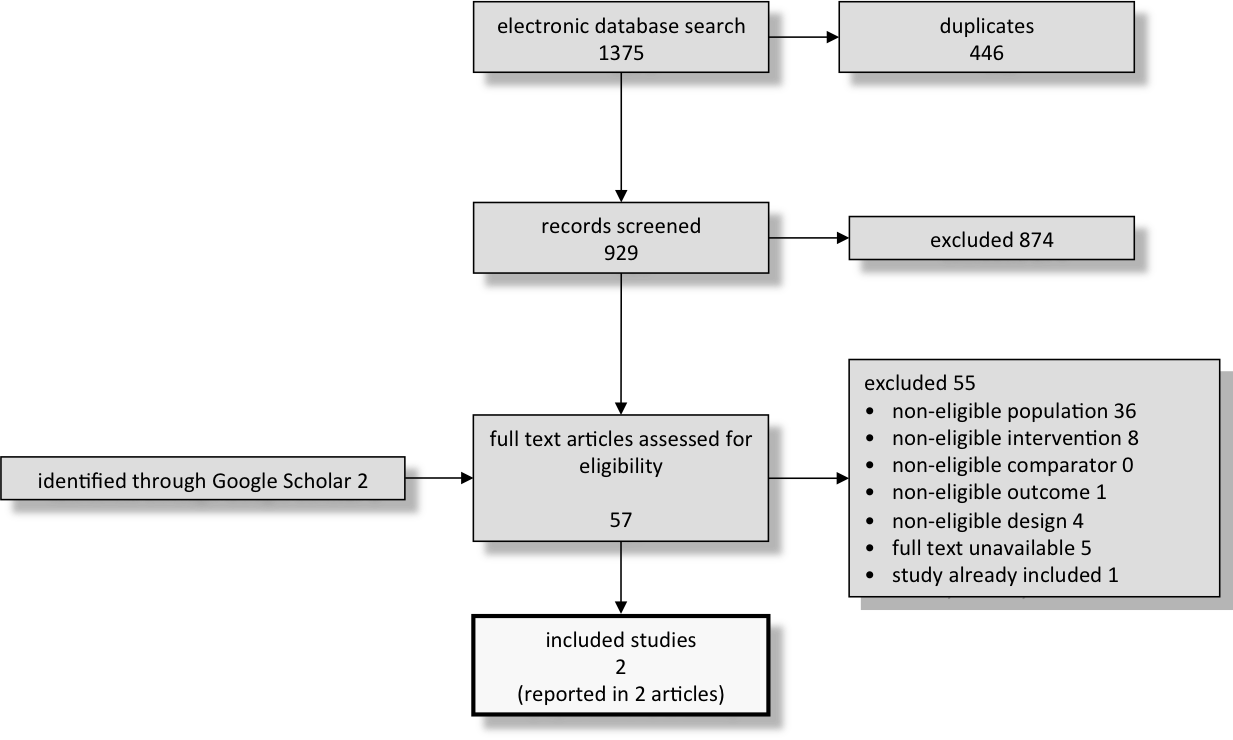
***

***C: Search update (2014)***

***
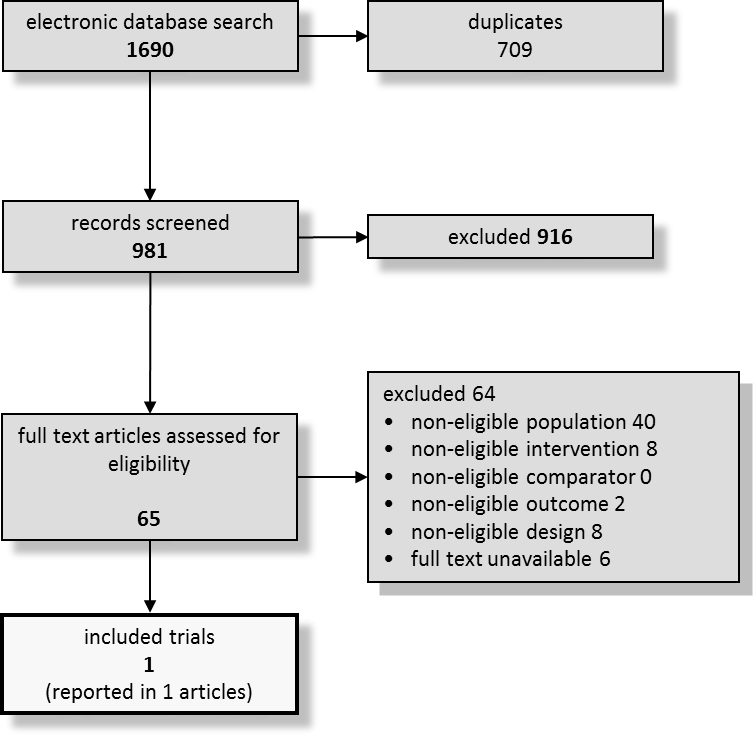
***

***D: Search update MEDLINE (2016)***


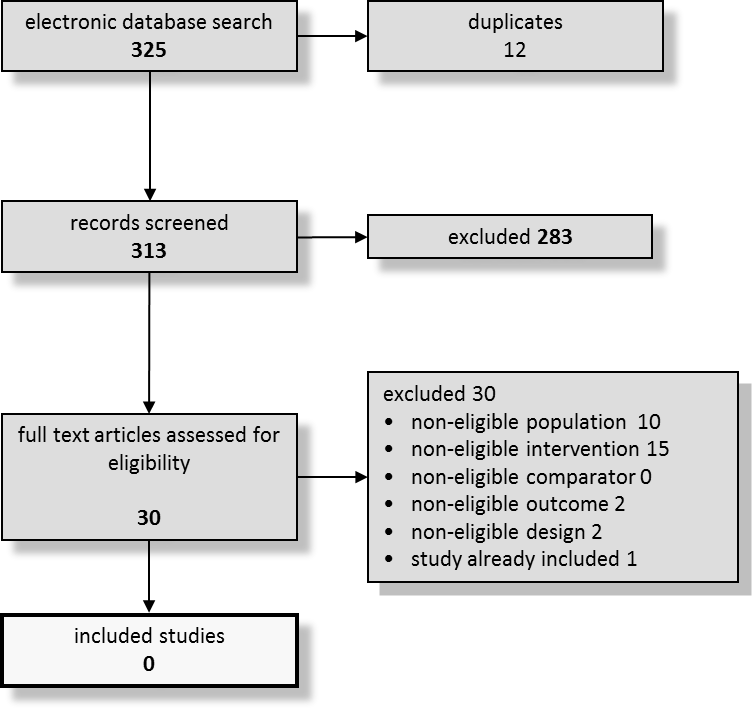

Supplement: S2 File — (DOCX) [file pone.0153380.s003.docx]
