# Supplementary material for: Comparative Safety of Pharmacologic Treatments for Persistent Depressive Disorder: A Systematic Review and Network Meta-Analysis
Source: PLoS One. 2016 May 17;11(5):e0153380. doi: 10.1371/journal.pone.0153380 (PMC4871495; doi:10.1371/journal.pone.0153380)
Supplement: S3 File — (DOCX) [file pone.0153380.s004.docx]

# S3 File. Complete reference list of included studies

**Aguglia 1995**

Aguglia E, de Vanna M, di Grazia MF. Role of Selective serotonin reuptake inhibitors on the treatment of chronic and/or resistant Major Depression. [Italian]. *Giornale Di Neuropsicofarmacologia.* 1995;17(3):73–77.

**Amore 2001**

Amore M, Jori MC. Faster response on amisulpride 50 mg versus sertraline 50-100 mg in patients with dysthymia or double depression: a randomized, double-blind, parallel group study. Amisert Investigators . *Int Clin Psychopharmacol.* 2001;16(6):317–324.

**Anisman 1999**

Anisman H, Ravindran AV, Griffiths J, Merali Z. Interleukin-1 beta production in dysthymia before and after pharmacotherapy. *Biol Psychiatry.* 1999;46(12):1649–1655.

**Bakish 1993**

Bakish D, Lapierre YD, Weinstein R, et al. Ritanserin, imipramine, and placebo in the treatment of dysthymic disorder. *J Clin Psychopharmacol.* 1993;13(6):409–414.

**Bella 1990/Fulgente 1990**

Bella R, Biondi R, Raffaele R, Pennisi G. Effect of acetyl-L-carnitine on geriatric patients suffering from dysthymic disorders. *Int J Clin Pharmacol Res.* 1990;10(6): 355–360. 9

Fulgente T, Onofrj M, Del Re ML, et al. Laevo-Acetylcarnitine Treatment of Senile Depression. *Clin Trials J.* 1990; 27(3):155–163.

**Bellino 1997**

Bellino S, Barzega G, Bogetto F, Maina G, Venturello S, Ravizza L. An open-label, randomized, prospective comparison of sertraline and amisulpride in the treatment of dysthymia in the elderly. *Curr Ther Res Clin Exp.* 1997; 58(10):798–808.

**Bersani 1991**

Bersani G, Pozzi F, Marini S, Grispini A, Pasini A, Ciani N. 5-HT2 receptor antagonism in dysthymic disorder: a double-blind placebo-controlled study with ritanserin. *Acta Psychiatr Scand.* 1991; 83(4):244–248.

**Bersani 2013**

Bersani G, Mecob G, Denaroe A, Liberatia D, Collettia C, Nicolaic R, Bersani F, Koverech F. L-Acetylcarnitineindysthymicdisorderin elderly patients: Adouble-blind, multicenter, controlled randomized study vs. Fluoxetine. Eur Neuropsychopharmacol. 2013; 23: 1219-1225.

**Bogetto 1997**

Bogetto F, Barzega G, Bellino S, Maina G, Ravizza L. Drug treatment of dysthymia: A clinical study. [Italian]. *Riv Psichiatr.* 1997;32(1):1–5.

**Boyer 1996a**

Boyer P, Lecrubier Y, Stalla-Bourdillon A, Fleurot O. Amisulpride versus amineptine and placebo for the treatment of dysthymia. *Neuropsychobiology.* 1999;39(1):25–32.

Boyer P, Lecrubier Y. Atypical antipsychotic drugs in dysthymia: placebo controlled studies of amisulpride versus imipramine, versus amineptine. *Eur Psychiatry.* 1996;11(3):135-140.

**Boyer 1996b**

Lecrubier Y, Boyer P, Turjanski S, Rein W, Amisulpride Study Group. Amisulpride versus imipramine and placebo in dysthymia and major depression. *J Affect Disord.* 1997;43(2):95-103.

Boyer P, Lecrubier Y. Atypical antipsychotic drugs in dysthymia: placebo controlled studies of amisulpride versus imipramine, versus amineptine. *Eur Psychiatry.* 1996;11(3):135-140.

**Devanand 2005**

Devanand DP, Nobler MS, Cheng J, et al. Randomized, double-blind, placebo-controlled trial of fluoxetine treatment for elderly patients with dysthymic disorder. *Am J Geriatr Psychiatry.* 2005;13(1):59–68.

**Duarte 1996**

Duarte A, Mikkelsen H, Delini-Stula A. Moclobemide versus fluoxetine for double depression: a randomized double-blind study. *J Psychiatr Res.* 1996;30(6):453–458.

**Geisler 1992**

Geisler A, Mygind S, Riis Knudsen O, Sloth-Nielsen M. Ritanserin and flupenthixol in dysthymic disorder. A controlled double-blind study in general practice. *Nord J Psychiatry.* 1992;46(4):237–243.

**Hellerstein 1993**

Hellerstein DJ, Yanowitch P, Rosenthal J, et al. A randomized double-blind study of fluoxetine versus placebo in the treatment of dysthymia. *Am J Psychiatry.* 1993;150(8):1169–1175.

**Hellerstein 1994/Rosenthal 1992**

Hellerstein DJ, Yanowitch P, Rosenthal J, et al. Long-term treatment of double depression: a preliminary study with serotonergic antidepressants. *Biol Psychiatry.* 1994;18(1):139–147.

Rosenthal J, Hemlock C, Hellerstein DJ, et al. A preliminary study of serotonergic antidepressants in treatment of dysthymia. *Prog Neuropsychopharmacol Biol Psychiatry.* 1992;16(6): 933–941.

**Hellerstein 2010**

Hellerstein DJ, Batchelder ST, Hyler S, et al. Escitalopram versus placebo in the treatment of dysthymic disorder. *Int Clin Psychopharmacol.* 2010;25(3):143–148.

**Hellerstein 2012**

Hellerstein DJ, Stewart JW, McGrath PJ, et al. A randomized controlled trial of duloxetine versus placebo in the treatment of nonmajor chronic depression. *J Clin Psychiatry.* 2012;73(7):984–991.

**Katona 1999**

Katona C, Bercoff E, Chiu E, Tack P, Versiani M, Woelk H. Reboxetine versus imipramine in the treatment of elderly patients with depressive disorders: a double-blind randomised trial. *J Affect Disord.* 1999;55(2–3): 203–213.

**Kocsis 1988**

Kocsis JH, Frances AJ, Voss C, Mann JJ, Mason BJ, Sweeney J. Imipramine treatment for chronic depression. *Arch Gen Psychiatry.* 1988;45(3):253–257.

**Leon 1994**

León CA, Vigoya J, Conde S, Campo G, Castrillón E, León A. Comparison of the effect of amisulpride and viloxazine in the treatment of dysthymia.[Spanish]. *Acta Psiquiatr Psicol Am Lat.* 1994;40(1):41–49.

**Ravindran 1999**

Ravindran AV, Anisman H, Merali Z, et al. Treatment of primary dysthymia with group cognitive therapy and pharmacotherapy: clinical symptoms and functional impairments. *Am J Psychiatry.* 1999;156(10):1608–1617.

**Ravindran 2000**

Ravindran AV, Guelfi JD, Lane RM, Cassano GB. Treatment of dysthymia with sertraline: a double-blind, placebo-controlled trial in dysthymic patients without major depression. *J Clin Psychiatry.* 2000;61(11):821–827.

**Ravindran 2013**

Ravindran AV, Cameron C, Bhatla R, Ravindran LN, da Silva TL. Paroxetine in the treatment of dysthymic disorder without co-morbidities: A double-blind, placebo-controlled, flexible-dose study. *Asian J Psychiatr.* 2013;6(2):157-161.

**Ravizza 1999**

Ravizza L. Amisulpride in medium-term treatment of dysthymia: a six-month, double-blind safety study versus amitriptyline. AMILONG investigators. *J Psychopharmacol.* 1999;13(3):248–254.

**Rocca 2002a**

Rocca P, Fonzo V, Ravizza L, et al. A comparison of paroxetine and amisulpride in the treatment of dysthymic disorder. *J Affect Disord.* 2002;70(3):313–317.

**Rush 1998/Keller 1998**

Rush AJ, Koran LM, Keller MB, et al. The treatment of chronic depression, part 1: study design and rationale for evaluating the comparative efficacy of sertraline and imipramine as acute, crossover, continuation, and maintenance phase therapies. *J Clin Psychiatry.* 1998; **59**: 589–97.

Keller MB, Gelenberg AJ, Hirschfeld RMA, et al. The treatment of chronic depression, part 2: a double-blind, randomized trial of sertraline and imipramine. *J Clin Psychiatry.* 1998;59(11):598–607.

**Salzmann 1995**

Salzmann E, Robin JL. Multicentric double-blind study comparing efficacy and safety of minaprine and imipramine in dysthymic disorders. *Neuropsychobiology*. 1995;31(2):68–75.

**Smeraldi 1998**

Smeraldi E. Amisulpride versus fluoxetine in patients with dysthymia or major depression in partial remission: a double-blind, comparative study. *J Affect Disord.* 1998;48(1):47–56.

**Thase 1996**

Thase ME, Fava M, Halbreich U, et al. A placebo-controlled, randomized clinical trial comparing sertraline and imipramine for the treatment of dysthymia. *Arch Gen Psychiatry.* 1996;53(9):777–784.

**Vallejo 1987**

Vallejo J, Gasto C, Catalan R, Salamero M. Double-blind study of imipramine versus phenelzine in melancholias and dysthymic disorders. *Br J Psychiatry.* 1987;151(5):639–642.

**Vanelle 1997**

Vanelle J-M, Attar-Levy D, Poirier M-F, Bouhassira M, Blin P, Olié J-P. Controlled efficacy study of fluoxetine in dysthymia. *Br J Psychiatry.* 1997;170(4):345–350.

**Versiani 1997**

Versiani M, Amrein R, Stabl M. Moclobemide and imipramine in chronic depression (dysthymia): an international double-blind, placebo-controlled trial. International Collaborative Study Group. *Int Clin Psychopharmacol.* 1997;12(4):183–193.

**Zanardi 2006**

Zanardi R, Smeraldi E. A double-blind, randomised, controlled clinical trial of acetyl-L-carnitine vs. amisulpride in the treatment of dysthymia. *Eur Neuropsychopharmacol.* 2006;16(4):281–287.
