# Supplementary material for: Comparative Safety of Pharmacologic Treatments for Persistent Depressive Disorder: A Systematic Review and Network Meta-Analysis
Source: PLoS One. 2016 May 17;11(5):e0153380. doi: 10.1371/journal.pone.0153380 (PMC4871495; doi:10.1371/journal.pone.0153380)
Supplement: S1 Table — (DOCX) [file pone.0153380.s005.docx]

# S1 Table. Items to assess the methodological quality of studies that examined adverse events

| **Items of the Cochrane Collaboration´s risk of bias tool** | **Items to assess the methodological quality of studies that examined adverse events** | **Commentary** |
| --- | --- | --- |
| Allocation sequence generation adequate | Allocation sequence generation adequate | Adopted from the risk of bias tool without modification |
| Allocation concealment adequate | Allocation concealment adequate | Adopted from the risk of bias tool without modification |
| Blinding adequate (patient and clinician) | Blinding adequate (patient and clinician) | Adopted from the risk of bias tool without modification |
| Blinding adequate (outcome assessment) | Blinding adequate (assessment of adverse events) | Adopted from the risk of bias tool (modified for adverse events) |
| Incomplete data adequately addressed | Exclusion of patients from adverse event analysis (safety sample) adequately addressed | Modified  *yes:* safety sample corresponds to randomized sample or adequate definition of safety sample reported (including reasons for exclusion), *no:* no adequate definition of safety sample reported and safety sample does not correspond to randomized sample, no analysis of individual adverse events (e.g. only dropout due to adverse event reported) |
| Free of selective outcome reporting | Transparent regarding selective reporting of adverse events | Modified  *yes:* all or defined sample of adverse events reported (e.g. adverse events reported by at least 2% of the patients), *no:* undefined or no sample of adverse events reported |
| **-** | Definition of adverse events reported | Extended  *yes:* any definition of specific adverse events reported, *no:* no definition of specific adverse events reported |
| **-** | Methods used for monitoring adverse events adequate | Extended  *yes:* checklist, Adverse event form or open question, *no:* unprompted patient report, spontaneous observation or retrospective chart view, *unclear:* not reported or unspecific information |
| Free of other bias | Free of other quality limitations | Adopted from the risk of bias tool without modification |
