# Supplementary material for: Comparative Safety of Pharmacologic Treatments for Persistent Depressive Disorder: A Systematic Review and Network Meta-Analysis
Source: PLoS One. 2016 May 17;11(5):e0153380. doi: 10.1371/journal.pone.0153380 (PMC4871495; doi:10.1371/journal.pone.0153380)
Supplement: S2 Table — (DOCX) [file pone.0153380.s006.docx]

# S2 Table. Selected characteristics of included studies

| study ID  *country* | relevant study arms | diagnosis | mean age of sample (years) | % female in sample | setting | duration in weeks | dosis (per day) | N  (safety sample) | N  (rand. sample) | rate of experiencing any AE | rate of discontinuing due to AE | assessment methods | included in analysis |
| --- | --- | --- | --- | --- | --- | --- | --- | --- | --- | --- | --- | --- | --- |
| Aguglia 1995  *Italy* | 1. clomipramine (TCA)  2. fluoxetine (SSRI) | cMD | 45.0 | n.a. | inpatient and outpatient | 4 | 75-150 mg  20-40 mg | 48 | 48 |  |  | unclear | A |
| Amore 2001  *Italy* | 1. amisulpride (aPS)  2. sertraline (SSRI) | Dys, DD | 47.1 | 68 | outpatient | 12 | 50 mg  50-100 mg | 313 | 313 | 70/157  74/156 | 10/157  12/156 | unclear | A, B, C |
| Anisman 1999  *Canada* | 1. sertraline (SSRI)  2. placebo | Dys | 40.5 | 52 | unclear | 12 | 100-200 mg | 68 | 68 |  | 0/34  0/34 | unclear | C |
| Bakish 1993  *Canada* | 1. imipramine (TCA)  2. ritanserin  (oAD)  3. placebo | Dys | 37.6 | 52 | outpatient | 7 | 50-200 mg  5-20 mg | 50 | 50 |  | 3/16  2/17  0/17 | open questions | C |
| Bella/ Fulgente 1990  *Italy* | 1. acetyl-l-carnitine (cmpl)  2. placebo | Dys | 75.0 | 58 | first inpatient, then outpatient | 8 | 3 g | 46 | 60 |  | 0/30  0/30 | AE checklist | C |
| Bellino 1997  *Italy* | 1. amilsulpride (aPS)  2. sertraline (SSRI) | Dys | 70.6 | 65 | outpatient | 24 | 50 mg  50 mg | 49 | 49 | 3/23  7/26 | 1/23  4/26 | unclear | A, B, C |
| Bersani 1991  *Italy* | 1. ritanserin  (oAD)  2. placebo | Dys | 42.5 | 57 | outpatient | 5 | 10 mg | 29 | 30 | 10/15  4/14 | 1/15  0/15 | unprompted patient reports | B, C |
| Bersani 2013  *Italy* | 1. acetyl-l-carnitine (cmpl)  2. fluoxetine (SSRI) | Dys | 71.7 | 74 | outpatient | 7 | 3 g  20 mg | 80 | 80 | 7/41  18/39 | 0/41  3/39 | AE scale | A, B, C |
| Bogetto 1997  *Italy* | 1. amisulpride (aPS)  2. fluoxetine (SSRI)  3. lorazepam (Benz) | Dys | 29.5 | 64 | outpatient | 8 | 50 mg  20 mg  3 mg | 39 | 39 | 4/12  6/14  4/13 |  | semi-  structured interview | A_,_ B |
| Boyer 1996 A  *France* | 1. amineptine (TCA)  2. amisulpride (aPS)  3. placebo | Dys | 48.0 | 77 | outpatient | 12 | 200 mg  50 mg | 323 | 323 | 69/111  57/104  48/108 | 9/111  7/104  4/108 | unprompted patient reports | B, C |
| Boyer 1996 B  *France* | 1. imipramine (TCA)  2. amisulpride (aPS)  3. placebo | Dys, DD, rec | 43.5 | 54 | outpatient | 24 | 100 mg  50 mg | 219 | 219 | 63/73  39/73  43/73 | 17/73  8/73  2/73 | open questions and AE scale | A, B, C |
| Devanand 2005  *USA* | 1. fluoxetine (SSRI)  2. placebo | Dys | 69.9 | 37 | outpatient | 12 | 20-60 mg | 90 | 90 |  | 3/44  1/46 | AE scale and clinical manual | C |
| Duarte 1996  *Argentina* | 1. moclobemide (MAO)  2. fluoxetine (SSRI) | DD | 45.9 | 41 | outpatient | 6 | 300 mg  200 mg | 42 | 42 |  | 0/21  0/21 | unprompted patient reports | A, C |
| Geisler 1992  *Denmark* | 1. ritanserin  (oAD)  2. flupenthixol (aPS) | Dys | 47.7 | 73 | outpatient | 6 | 5-10 mg  1-2 mg | 69 | 70 | 15/33  16/36 | 2/33  2/37 | AE scale | A, B, C |
| Hellerstein 1993  *USA* | 1. fluoxetine (SSRI)  2. placebo | Dys | 36.2 | 50 | outpatient | 8 | 20-60 mg | 35 | 35 |  | 1/19  0/16 | retrospective chart view | A, C |
| Rosenthal 1992/ Hellerstein 1994  *USA* | 1. fluoxetine (SSRI)  2. trazodone  (SARI) | Dys, DD | 40.2 | 74 | outpatient | 12 | 20-60 mg  50-350 mg | 38 | 38 |  | 1/20  7/18 | unclear | C |
| Hellerstein 2012  *USA* | 1. duloxetine (SNRI)  2. placebo | Dys | 41.6 | 41 | outpatient | 10 | 30-120 mg | 57 | 57 | 27/29  22/28 |  | open questions | A_,_ B |
| Hellerstein 2010  *USA* | 1. escitalopram (SSRI)  2. placebo | Dys | 44.7 | 50 | outpatient | 12 | 10-20 mg | 32 | 34 | 15/17  14/15 | 1/17  0/17 | open questions | A, B, C |
| Katona 1999  *mc^a^* | 1. reboxetin (SNRI)  2. imipramine (TCA) | Dys | 74.2 | 76 | inpatient and outpatient | 8 | 4-6 mg  50-100 mg | 129 | 129 | 48/67  42/62 |  | AE checklist and open questions | A_,_ B, |
| Kocsis 1988  *USA* | 1. imipramine (TCA)  2. placebo | Dys, DD | 39.0 | 70 | outpatient | 6 | 50-300 mg | 54 | 54 |  | 2/29  0/25 | AE checklist | C |
| León 1994  *Columbia* | 1. amisulpride (aPS)  2. viloxazin (SNRI) | Dys | 32.1 | 94 | outpatient | 4 | 50 mg  50-150 mg | 78 | 80 |  |  | AE scale and unprompted patient reports | A |
| Ravindran 1999  *Canada* | 1. sertraline (SSRI)  2. placebo | Dys | 38.0 | 58 | unclear | 12 | 50-200 mg | 48 | 48 |  | 0/22  0/26 | unclear | C |
| Ravindran 2000  *mc^b^* | 1. sertraline (SSRI)  2. placebo | Dys | 45.1 | 67 | outpatient | 12 | 50-200 mg | 310 | 310 | 119/158  98/152 | 21/158  12/152 | unprompted patient report and clinical observations | A, B, C |
| Ravindran 2013  *Canada* | 1. paroxetine (SSRI)  2. placebo | Dys | 41.7 | 48 | outpatient | 12 | 20-40 mg | 40 | 40 | 17/21  11/19 | 0/21  0/19 | unclear | A, B, C |
| Ravizza 1999  *Italy* | 1. amisulpride (aPS)  2. amitriptylin (TCA) | Dys, cMD | 47.1 | 64 | outpatient | 24 | 50 mg  25-75 mg | 250 | 253 | 106/165  62/85 | 23/166  11/87 | AE scale | A, B, C |
| Rocca 2002 a  *Italy* | 1. paroxetine (SSRI)  2. amilsulpride (aPS) | Dys | 45.0 | 70 | outpatient | 6 | 20 mg  50 mg | 118 | 118 |  | 6/70  4/48 | unclear | C |
| Rush/ Keller 1998  *USA* | 1. imipramine (TCA)  2. sertraline (SSRI) | Dys, DD | 41.1 | 63 | outpatient | 12 | 50-300 mg  50-200 mg | 635 | 635 | 25/209  27/426 |  | unprompted patient report and clinical observations | A, C |
| Salzmann 1995  *Germany* | 1. minaprine (oAD)  2. imipramine (TCA) | Dys | 55.1 | 81 | outpatient | 6 | 200 mg  50-100 mg | 67 | 67 | 14/33  20/34 | 3/33  4/34 | AE scale and open questions | A, B, C |
| Smeraldi 1998  *Italy* | 1. amisulpride (aPS)  2. fluoxetine (SSRI) | Dys, MD in partial remission | 49.4 | 68 | outpatient | 12 | 50 mg  20 mg | 278 | 281 | 67/141  56/137 | 13/142  10/139 | AE scale | A, B, C |
| Thase 1996  *USA* | 1. imipramine (TCA)  2. sertraline (SSRI)  3. placebo | Dys | 41.7 | 65 | outpatient | 12 | 50-300 mg  50-200 mg | 410 | 410 |  | 28/136  11/134  9/140 | unclear | A, C |
| Vallejo 1987  *Spain* | 1. imipramine (TCA)  2. phenelzine (MAO) | Dys | 40.2 | 81 | outpatient | 6 | 100-250 mg  30-75 mg | 32 | 32 |  | 4/20  3/19 | unclear | C |
| Vanelle 1997  *France* | 1. fluoxetine (SSRI)  2. placebo | Dys | 43.0 | 76 | inpatient and outpatient | 12 | 20 mg | 140 | 140 | 35/91  22/49 |  | unclear | A _,_ B |
| Versiani 1997  *mc^c^* | 1. imipramine (TCA)  2. moclobemide (MAO)  3. placebo | Dys, DD | 41.5 | 70 | outpatient | 8 | 25-250 mg  75-750 mg | 315 | 315 | 83/103  73/108  61/104 | 11/103  8/108  2/104 | unclear | A, B, C |
| Zanardi 2006  *Italy* | 1. acetyl-l-carnitine (cmpl)  2. amisulpride (aPS) | Dys | 47.4 | 68 | outpatient | 12 | 500 mg  50 mg | 204 | 204 | 10/105  29/99 | 3/105  21/99 | unprompted patient reports | A, B, C |

mc=multicenter; ^a^Australia, Belgium, Brazil, France, Germany, Ireland, UK; ^b^Canada, France, Italy, Spain, Sweden, UK; ^c^Argentina, Brazil, Chile; cMD=chronic major depression; Dys=dysthymy; DD=double epression; rec=recurrent major depression with incomplete remission between episodes; SSRI=selective serotonine reuptake inhibitors; SNRI=serotonine noradrenaline reuptake inhibitor; SARI=serotonine antagonist and reuptake inhibitor; TCA=trycyclic antidepressants; MAOI=monoaminoxidase inhibitor; aPS=antipsychotic; cmpl=complementary treatment; Benz=benzodiazepine; oAD=other antidepressant; n.a.=not available; N=sample size; A=included in (meta-) analyses for individual adverse events, B=included in network meta-analysis on the number of patients experiencing any adverse event; C=included in network meta-analysis on discontinuation due to adverse events.
