# Supplementary material for: Comparative Safety of Pharmacologic Treatments for Persistent Depressive Disorder: A Systematic Review and Network Meta-Analysis
Source: PLoS One. 2016 May 17;11(5):e0153380. doi: 10.1371/journal.pone.0153380 (PMC4871495; doi:10.1371/journal.pone.0153380)
Supplement: S3 Table — (DOCX) [file pone.0153380.s007.docx]

# S3 Table. Methodological quality in individual studies

|  | **Allocation sequation generation adequate** | **Allocation concealment adequate** | **Blinding adequate (patient and clinician)** | **Blinding adequate (AE assessment)** | **Exclusion from AE analysis adequately addressed** | **Transparency regarding selective reporting of AEs** | **Definition of AEs reported** | **Methods used for monitoring AEs adequate** | **Free of other quality limitations** | **Global methodological quality** |
| --- | --- | --- | --- | --- | --- | --- | --- | --- | --- | --- |
| Aguglia 1995 | unclear | unclear | yes | unclear | yes | yes | no | unclear | no | low |
| Amore  2001 | unclear | unclear | yes | unclear | yes | no | no | unclear | yes | low |
| Anisman 1999 | yes | unclear | yes | unclear | no | no | no | unclear | yes | low |
| Bakish  1993 | unclear | unclear | yes | unclear | no | no | no | yes | unclear | low |
| Bella/ Fulgente 1990 | unclear | unclear | unclear | unclear | no | no | no | yes | unclear | low |
| Bellino  1997 | yes | unclear | unclear | yes | yes | no | no | unclear | yes | unclear |
| Bersani 1991 | unclear | unclear | yes | unclear | yes | yes | no | no | yes | unclear |
| Bersani 2013 | yes | unclear | yes | unclear | yes | yes | no | yes | no | unclear |
| Bogetto 1997 | unclear | unclear | unclear | unclear | yes | no | no | yes | unclear | low |
| Boyer  1996 A | unclear | unclear | yes | unclear | yes | yes | no | no | yes | unclear |
| Boyer  1996 B | unclear | unclear | yes | unclear | yes | yes | no | yes | yes | unclear |
| Devanand 2005 | yes | yes | yes | yes | yes | yes | no | yes | yes | high |
| Duarte  1996 | unclear | unclear | yes | unclear | yes | yes | no | no | yes | unclear |
| Geisler 1992 | unclear | unclear | yes | unclear | no | yes | no | yes | yes | unclear |
| Hellerstein 1993 | unclear | unclear | yes | unclear | yes | yes | no | no | yes | unclear |
| Hellerstein 1994/ Rosenthal 1992 | unclear | unclear | no | no | no | no | no | unclear | yes | low |
| Hellerstein 2010 | unclear | unclear | yes | unclear | no | yes | no | yes | yes | unclear |
| Hellerstein 2012 | yes | unclear | yes | unclear | yes | no | no | yes | unclear | unclear |
| Katona 1999 | unclear | unclear | yes | unclear | yes | yes | no | yes | yes | high |
| Kocsis  1988 | unclear | unclear | yes | unclear | no | no | no | yes | unclear | low |
| León  1994 | unclear | unclear | yes | unclear | yes | yes | no | yes | yes | unclear |
| Ravindran 1999 | yes | unclear | yes | unclear | no | no | no | unclear | yes | low |
| Ravindran 2000 | unclear | unclear | yes | unclear | yes | yes | no | unclear | yes | unclear |
| Ravindran 2013 | unclear | unclear | yes | unclear | yes | yes | no | unclear | yes | unclear |
| Ravizza 1999 | unclear | unclear | yes | unclear | yes | yes | no | yes | yes | high |
| Rocca  2002 A | unclear | unclear | no | no | no | no | no | unclear | yes | low |
| Rush/ Keller 1998 | unclear | unclear | yes | unclear | yes | yes | no | no | yes | unclear |
| Salzmann 1995 | yes | unclear | yes | unclear | yes | yes | no | yes | yes | high |
| Smerladi 1998 | unclear | unclear | yes | unclear | yes | yes | no | yes | yes | unclear |
| Thase  1996 | yes | unclear | yes | unclear | yes | yes | no | unclear | unclear | unclear |
| Vallejo  1987 | unclear | unclear | yes | unclear | no | no | no | unclear | yes | low |
| Vanelle 1997 | unclear | unclear | yes | unclear | yes | no | no | unclear | yes | low |
| Versiani 1997 | unclear | unclear | yes | unclear | yes | yes | no | unclear | yes | unclear |
| Zanardi 2006 | unclear | unclear | yes | unclear | yes | yes | no | no | unclear | low |
| Summary “yes” | 8/34 | 1/34 | 29/34 | 2/34 | 24/34 | 21/34 | 0/34 | 15/34 | 25/34 | 5/34 |

high methodological quality = at least six of the criteria were met; low methodological quality = six or more of the criteria were not met.
