# Supplementary material for: Comparative Safety of Pharmacologic Treatments for Persistent Depressive Disorder: A Systematic Review and Network Meta-Analysis
Source: PLoS One. 2016 May 17;11(5):e0153380. doi: 10.1371/journal.pone.0153380 (PMC4871495; doi:10.1371/journal.pone.0153380)
Supplement: S4 Table — (DOCX) [file pone.0153380.s008.docx]

# S4 Table. Complete adverse event profiles for all investigated agents

|  | | **TCA** | | | **aPS** | | **SNRI** | | | **SSRI** | | | | **MAOI** | **Benz** | **Cmpl** | **oAD** | |
| --- | --- | --- | --- | --- | --- | --- | --- | --- | --- | --- | --- | --- | --- | --- | --- | --- | --- | --- |
| **Adverse Event** | | **IMI** | **AMT** | **CLO** | **AMS** | **FLP** | **DUL** | **REB** | **VIL** | **SER** | **PAR** | **FLO** | **ESC** | **MOC** | **LOR** | **ALC** | **RIT** | **MIN** |
| ***Gastrointestinal events and weight changes*** | |  |  |  |  |  |  |  |  |  |  |  |  |  |  |  |  |  |
|  | Gastrointestinal events |  | ⯃⯃ |  | ⯌⯌ |  |  |  |  | ⯃⯃ |  |  |  |  |  |  |  |  |
|  | Gastrointestinal upset |  |  |  |  |  | ⯌ |  |  |  |  |  | ⯌ |  |  |  |  |  |
|  | Constipation | ⯃⯃ | ⯃⯃ | ⯃⯃ | 🞿⯌ |  | ⯃ | ⯌⯌ | ⯃⯃ | ⯃⯌ |  | ⯌⯌ |  | ⯌⯌ |  |  |  |  |
|  | Vomiting | ⯃⯃ |  |  | ⯌⯌ | ⯃⯃ |  |  |  | ⯃⯌ |  | ⯃⯃ |  |  |  | ⯌⯌ | ⯌⯌ |  |
|  | Nausea | 🞿🞿 |  |  | 🞿⯌ | ⯃⯃ | ⯃ | ⯌⯌ |  | ⯃⯃ | ⯃ | ⯃⯃ |  | ⯃⯃ |  | ⯌⯌ | ⯌⯌ |  |
|  | Diarrhea | ⯌⯌ |  |  |  | ⯌⯌ |  |  |  | ⯃⯃ | ⯃ |  |  |  |  | ⯌⯌ | ⯃⯃ |  |
|  | Dyspepsia |  |  |  | 🞿🞿 | ⯃⯃ |  |  |  | ⯃🞿 |  | ⯃⯃ |  |  |  | ⯃⯃ | ⯌⯌ |  |
|  | Abdominal pain |  |  |  | ⯃⯃ |  |  |  |  | ⯃ |  | ⯌⯌ |  |  |  |  |  |  |
|  | Flatulence |  |  |  |  |  |  |  |  | ⯃ |  |  |  |  |  |  |  |  |
|  | Borborygmi |  |  |  |  | ⯌⯌ |  |  |  |  |  |  |  |  |  |  | ⯃⯃ |  |
|  | Anorexia | ⯃⯌ |  |  | ⯌⯌ |  |  |  |  | ⯃⯃ |  | ⯃⯃ |  |  |  | ⯌⯌ |  |  |
|  | Weight loss |  |  |  |  |  |  |  |  |  |  |  |  |  |  |  | ⯃⯃ |  |
|  | Increased appetite | ⯃ |  |  | ⯃⯃ |  |  |  |  | ⯌⯌ |  | ⯌⯌ |  |  |  |  |  |  |
|  | Decreased appetite |  |  |  |  |  | ⯃ |  |  |  |  |  |  |  |  |  |  |  |
|  | Weight gain |  | ⯌⯌ |  | ⯃⯃ | ⯃⯃ |  |  | ⯌⯌ |  |  | ⯌⯌ |  |  | ⯃⯃ | ⯌⯌ | ⯌⯌ |  |
|  | Changes in weight |  |  |  |  | ⯌⯌ |  |  |  |  |  |  |  |  |  |  |  |  |
| ***Activating/ sedating events*** | |  |  |  |  |  |  |  |  |  |  |  |  |  |  |  |  |  |
|  | Sleepiness/ Somnolence/ Hypersomnia | ⯃⯃ | ⯃⯃ |  | 🞿⯌ | ⯌⯌ |  | ⯌⯌ | ⯌⯌ | ⯃⯌ |  | ⯌⯌ |  | ⯌⯌ | ⯃⯃ | ⯌⯌ | ⯃⯃ |  |
|  | Increased duration of sleep |  |  |  |  | ⯌⯌ |  |  |  |  |  |  |  |  |  |  | ⯃⯃ |  |
|  | Insomnia/ Reduced duration of sleep | ⯌⯌ |  |  | 🞿🞿 | ⯃⯃ | ⯃ | ⯃⯃ | ⯃⯃ | ⯃⯃ |  | ⯃⯃ |  | ⯃⯃ |  | ⯌⯌ | ⯌⯌ |  |
|  | Insufficient sleep |  |  |  | ⯌⯌ |  |  |  | ⯃⯃ |  |  |  |  |  |  |  |  |  |
|  | Agitation/ Tension | ⯃⯌ |  |  | ⯌⯌ | ⯌⯌ | ⯃ | ⯃⯃ |  | ⯃⯃ |  | ⯃⯃ |  |  |  | ⯌⯌ |  |  |
|  | Nervousness/ Anxiety | ⯃⯃ |  |  | 🞿🞿 |  | ⯃ |  |  | ⯃⯌ |  | 🞿🞿 |  | ⯃⯃ |  |  |  |  |
|  | Asthenia/ Fatigue | ⯃⯃ | ⯃⯃ |  | 🞿⯌ | ⯌⯌ | ⯃ | ⯌⯌ |  | ⯃ | ⯃ | ⯌⯌ |  |  | ⯃⯃ | ⯌⯌ | ⯃⯃ |  |
|  | Vivid dreams/ Increased dream activity |  |  |  |  | ⯌⯌ | ⯃ |  |  |  |  |  |  |  |  |  | ⯃⯃ |  |
|  | Reduction in motor activity |  |  |  |  |  |  |  |  |  |  | ⯌⯌ |  |  |  | ⯃⯃ |  |  |
|  | Increase in motor activity |  |  |  |  |  |  |  |  |  |  | ⯃⯃ |  |  |  | ⯌⯌ |  |  |
| ***Emotional/ psychic events*** | |  |  |  |  |  |  |  |  |  |  |  |  |  |  |  |  |  |
|  | Psychic events |  |  |  |  | ⯌⯌ |  |  |  |  |  |  |  |  |  |  | ⯃⯃ |  |
|  | Emotional indifferences |  |  |  |  | ⯌⯌ |  |  |  |  |  |  |  |  |  |  | ⯃⯃ |  |
|  | Depression |  |  |  |  | ⯃⯃ |  |  |  |  |  | ⯌⯌ |  |  |  |  | ⯌⯌ |  |
| ***(Anti-)cholinergic events and headache*** | |  |  |  |  |  |  |  |  |  |  |  |  |  |  |  |  |  |
|  | Dry mouth | ⯃⯃  ⯌⯌ | ⯃⯃ | ⯃⯃ | ⯃⯌ |  | ⯃ | ⯃⯃ | ⯃⯃ | ⯃⯌ |  | ⯌⯌ |  | ⯃⯌ |  | ⯌⯌ |  |  |
|  | Excessive thirst | ⯃⯃ |  |  | ⯌⯌ |  |  |  |  |  |  |  |  |  |  |  |  |  |
|  | Increased salivation |  |  |  |  | ⯃⯃ |  |  |  |  |  |  |  |  |  |  | ⯌⯌ |  |
|  | Reduced salivation |  |  |  |  | ⯌⯌ |  |  |  |  |  |  |  |  |  |  | ⯃⯃ |  |
|  | Taste perversion/ Bitter taste |  | ⯃⯃ |  | ⯌⯌ |  |  |  | ⯃⯃ |  |  |  |  |  |  |  |  |  |
|  | Blurred/ abnormal vision | ⯃⯃ |  |  | ⯃⯌ | ⯃⯃ |  | ⯌⯌ |  | ⯃⯌ |  | ⯃⯃ |  | ⯃⯌ |  | ⯌⯌ | ⯌⯌ |  |
|  | Sweating | ⯃⯃ | ⯃⯃ |  | ⯌⯌ |  |  |  |  | ⯃⯌ |  |  |  |  |  | ⯌⯌ |  |  |
|  | Hot flushes |  | ⯃⯃ |  | ⯃⯃  ⯌⯌ |  |  |  | ⯌⯌ |  |  |  |  |  |  |  |  |  |
|  | Headache/ Lightheadedness | ⯌⯌ | ⯃⯃ | ⯌⯌ | 🞿🞿 | 🞿🞿 | ⯃ | ⯃⯃ | ⯌⯌ | ⯌⯃ | ⯃ | 🞿🞿 |  | ⯃⯃ |  | ⯌⯌ | 🞿🞿 |  |
|  | Sweating/ diaphoresis |  |  | ⯃⯃ |  |  |  | ⯌⯌ |  |  | ⯃ | 🞿🞿 |  | ⯃ |  |  | ⯌⯌ |  |
|  | Dizziness | ⯃⯃ | ⯃⯃ |  | 🞿⯌ | ⯌⯌ | ⯌ |  | ⯃⯃ | ⯃⯌ |  | ⯌⯌ |  | ⯃⯌ | ⯃⯃ | ⯌⯌ | ⯃⯃ |  |
|  | Micturition difficulties |  |  |  | ⯃ | ⯌⯌ |  |  |  | ⯃ |  |  |  |  |  |  | ⯃⯃ |  |
|  | Polyuria |  |  |  |  | ⯃⯃ |  |  |  |  |  |  |  |  |  |  | ⯌⯌ |  |
|  | Events of the central nervous system |  | ⯃⯃ |  | ⯌⯌ |  |  |  |  |  |  |  |  |  |  |  |  |  |
|  | Events of the autonomic nervous system | ⯃⯃ | ⯃⯃ |  | ⯌⯌ | ⯌⯌ |  |  |  |  |  |  |  |  |  |  | ⯃⯃ | ⯌⯌ |
|  | Neurological events | ⯃⯃ | ⯃⯃ |  | ⯃⯌ | ⯌⯌ |  |  |  |  |  | ⯃⯃ |  |  |  |  | ⯃⯃ |  |
| ***Extrapyramidal events*** | |  |  |  |  |  |  |  |  |  |  |  |  |  |  |  |  |  |
|  | Tremor | ⯃⯃  ⯌⯌ | ⯃⯃ |  | ⯃⯃  ⯌⯌ | ⯌⯌ |  | ⯃⯃ |  | ⯃⯌ |  |  |  | ⯃⯌ |  | ⯌⯌ | ⯃⯃ |  |
|  | Dystonia |  |  |  |  | ⯃⯃ |  |  |  |  |  |  |  |  |  |  | ⯌⯌ |  |
|  | Hyperkinesia |  |  |  |  | ⯌⯌ |  |  |  |  |  |  |  |  |  |  | ⯃⯃ |  |
|  | Akathisia |  |  |  |  |  |  |  |  |  |  | ⯃⯃ |  |  |  | ⯌⯌ |  |  |
| ***Endocrine events*** | |  |  |  |  |  |  |  |  |  |  |  |  |  |  |  |  |  |
|  | Endocrine events | ⯃⯌ | ⯌⯌ |  | ⯃⯃ |  |  |  |  | ⯌⯌ |  |  |  |  |  |  |  |  |
|  | Sexual dysfunction | ⯃⯌ |  |  |  |  | ⯌ |  |  | ⯃⯃ | ⯃ | ⯃ |  |  |  |  |  |  |
|  | Galactorrhea/ Lactation |  | ⯌⯌ |  | ⯃⯃ |  |  |  | ⯌⯌ |  |  | ⯌⯌ |  |  |  | ⯌⯌ |  |  |
|  | Amenorrhea |  | ⯌⯌ |  | 🞿🞿 | ⯃⯃ |  |  | ⯃⯃ |  |  | ⯌⯌ |  |  | ⯃⯃ |  | ⯌⯌ |  |
|  | Libido reduction |  |  |  | ⯃⯃ |  | ⯌ |  |  |  |  | ⯃⯃  ⯌⯌ | ⯃ |  | ⯌⯌ |  |  |  |
|  | Increased sexual desire |  |  |  |  | ⯌⯌ |  |  |  |  |  |  |  |  |  |  | ⯃⯃ |  |
|  | Increased prolactin |  |  |  | ⯃⯃ |  |  |  |  |  |  |  |  |  |  | ⯌⯌ |  |  |
|  | Delayed orgasm |  |  |  |  |  | ⯃ |  |  |  |  |  |  |  |  |  |  |  |
| ***Dermatologic events*** | |  |  |  |  |  |  |  |  |  |  |  |  |  |  |  |  |  |
|  | Dermatologic events |  |  |  |  |  |  |  |  |  |  | ⯌⯌ |  |  |  |  |  |  |
|  | Rash | ⯃⯃ |  |  |  | ⯌⯌ | ⯃ |  |  | ⯃⯌ |  |  |  |  |  |  | ⯃⯃ |  |
|  | Flushing | ⯃⯃ |  |  |  |  |  |  |  | ⯌⯌ |  |  |  |  |  |  | ⯃⯃ |  |
|  | Pruritus |  |  |  |  | ⯌⯌ |  |  |  |  |  |  |  |  |  |  | ⯃⯃ |  |
| ***Cardiovascular events*** | |  |  |  |  |  |  |  |  |  |  |  |  |  |  |  |  |  |
|  | Hypotension | ⯃⯃ |  | ⯃⯃ |  |  |  | ⯌⯌ |  | ⯃⯌ |  | 🞿🞿 |  |  |  | ⯌⯌ |  |  |
|  | Hypertension |  |  |  |  |  |  |  |  |  |  | ⯌⯌ |  |  |  | ⯃⯃ |  |  |
|  | Palpitation | ⯃⯃ |  |  | ⯌⯌ | ⯌⯌ | ⯌ | ⯌⯌ |  | ⯃⯌ |  |  |  |  |  | ⯌⯌ | ⯃⯃ |  |
| ***Other events*** | |  |  |  |  |  |  |  |  |  |  |  |  |  |  |  |  |  |
|  | Influenza like events |  |  |  |  |  |  |  |  | ⯃ |  |  |  |  |  |  |  |  |
|  | Nasal congestion |  |  |  |  |  |  |  |  |  |  | ⯌⯌ |  |  |  |  |  |  |
|  | Pharyngitis |  |  |  |  |  |  |  |  | ⯃ |  |  |  |  |  |  |  |  |
|  | Upper respiratory tract infection |  |  |  |  |  |  |  |  | ⯃ |  |  |  |  |  |  |  |  |
|  | Muscle/ joint pain |  |  |  |  |  |  |  |  |  |  | ⯃ |  |  |  |  |  |  |
|  | Breast pain |  | ⯌⯌ |  | ⯃⯃ |  |  |  |  |  |  |  |  |  |  |  |  |  |
|  | Back pain |  |  |  |  |  |  |  |  | ⯌ |  |  |  |  |  |  |  |  |
|  | Edema |  |  |  | ⯃⯃ |  |  |  |  |  |  |  |  |  |  | ⯌⯌ |  |  |
|  | Confusion | ⯌⯌ |  |  |  |  |  | ⯃⯃ |  |  |  |  |  |  |  |  |  |  |
|  | Failing memory |  |  |  |  | ⯌⯌ |  |  |  |  |  |  |  |  |  |  | ⯃⯃ |  |
|  | Concentration difficulties |  |  |  |  | ⯌⯌ | ⯌ |  |  |  |  |  |  |  |  |  | ⯃⯃ |  |
|  | Paresthesia | 🞿🞿 |  |  |  |  |  | ⯃⯃ |  | ⯃⯌ |  |  |  |  |  |  |  |  |
|  | Rigidity |  |  |  |  | ⯌⯌ |  |  |  |  |  |  |  |  |  |  | ⯃⯃ |  |

TCA=tricyclic antidepressant; aPS=antipsychotic; SNRI=serotonin and norepinephrine reuptake inhibitor; SSRI=selective serotonin reuptake inhibitor; MAOI=monoamine oxidase inhibitor; Benz=benzodiazepine; Cmpl=complementary treatment; oAD=other antidepressants; IMI=imipramine; AMT=amitriptyline; CLO=clomipramine; AMS=amisulpride; FLP=flupenthixol; DUL=duloxetine; REB=reboxetine; VIL=viloxazine; SER=sertraline; PAR=paroxetine; FLO=fluoxetine; ESC=escitalopram; MOC=moclobemide; MIN=minaprine; LOR=lorazepam; RIT=ritanserin; ALC=acetyl-l-carnitine; ⯃⯃=significantly higher rate than at least one other agent; ⯃=significantly higher rate than placebo; ⯃⯃=significantly higher rate than placebo and non-significantly higher rate than at least one other agent; ⯌⯌=significantly lower rate than at least one other agent; ⯃⯃=non-significantly higher rate than at least one other agent; ⯃=non-significantly higher rate than placebo; ⯌=non-significantly lower rate than placebo; ⯌⯌=non-significantly lower rate than at least one other agent; ⯃⯌=significantly higher rate than placebo, but lower rate than at least one other agent; ⯃⯌=non-significantly higher rate than placebo, but lower rate than at least one other agent; ⯃⯌=non-significantly higher rate than placebo, but significantly lower rate than at least one other agent; ⯃⯌=significantly higher rate than placebo, but non-significantly lower rate than at least one other agent; 🞿🞿=non-significantly inconsistent results; 🞿⯌=significantly lower rate than at least one other agent, but also non-significantly inconsistent results; ⯃🞿=significantly higher rate than placebo, but also non-significantly inconsistent results; ⯌⯌=non-significantly lower rate than placebo, but significantly lower rate than at least one other agent; empty cells = comparative evidence is missing.
