# Supplementary material for: Comparative Safety of Pharmacologic Treatments for Persistent Depressive Disorder: A Systematic Review and Network Meta-Analysis
Source: PLoS One. 2016 May 17;11(5):e0153380. doi: 10.1371/journal.pone.0153380 (PMC4871495; doi:10.1371/journal.pone.0153380)
Supplement: S5 Table — (DOCX) [file pone.0153380.s009.docx]

# S5 Table. Odds ratios and corresponding 95% confidence intervals for all comparisons of agents with each other and with placebo

| **Adverse Event, by agent** | **Comparator** | **Number of included studies** | **Number of**  **included patients** | **OR (95% CI)** | **I^2^ (%)** |
| --- | --- | --- | --- | --- | --- |
| **Imipramine (TCA)** |  |  |  |  |  |
| Vomiting | Placebo  Sertraline | 1 (Thase 96)  1 (Thase 96) | 276  270 | 5.48 [1.18-25.48]  1.69 [0.60-4.80] |  |
| Constipation | Placebo  Sertraline  Moclobemide  Amisulpride  Reboxetine | 3 (Boyer 96B, Thase 96, Versiani 97)  2 (Thase 96, Rush/Keller 98)  1 (Versiani 97)  1 (Boyer 96B)  1 (Katona 99) | 629  905  211  146  129 | 3.14 [1.34-7.39]  3.89 [2.78-5.45]  2.54 [1.17-5.51]  3.37 [1.62-7.00]  1.39 [0.53-3.62] | 77.7  0 |
| Nausea | Placebo  Reboxetine | 2 (Thase 96, Versiani 97)  1 (Katona 99) | 483  129 | 1.44 [0.90-2.32]  1.09 [0.40-2.96] | 0 |
| Dyspepsia | Placebo  Sertraline | 1 (Thase 96)  2 (Thase 96, Rush/ Keller 98) | 276  905 | 2.08 [0.89-4.84]  1.21 [0.84-1.75] | 0 |
| Increased appetite | Sertraline | 1 (Thase 96) | 270 | 2.73 [0.71-10.52] |  |
| Sleepiness | Placebo  Sertraline  Moclobemide  Reboxetine | 2 (Versiani 97, Thase 96)  2 (Thase 96, Rush/Keller 98)  1 (Versiani 97)  1 (Katona 99) | 483  905  211  129 | 3.39 [2.11-5.45]  1.51 [1.11-2.07]  3.44 [1.65-7.17]  7.94 [0.40-156.97] | 0  0 |
| Nervousness/ Anxiety | Placebo  Sertraline | 1 (Thase 96)  2 (Thase 96, Rush/ Keller 98) | 276  905 | 1.45 [0.57-3.73]  1.28 [0.82-2.01] |  |
| Agitation/ Tension | Placebo | 1 (Thase 96) | 276 | 4.02 [1.10-14.74] |  |
| Asthenia/ Fatigue | Reboxetine | 1 (Katona 99) | 129 | 3.36 [0.34-33.15] |  |
| Anorexia | Placebo | 1 (Thase 96) | 276 | 4.21 [1.36-13.05] |  |
| Increased appetite | Placebo | 1 (Thase 96) | 276 | 1.69 [0.54-5.29] |  |
| Dry mouth | Placebo  Moclobemide  Sertraline  Amisulpride | 3 (Thase 96, Boyer 96B, Versiani 97)  1 (Versiani 97)  2 (Thase 96, Rush/ Keller 98)  1 (Boyer 96B) | 629  211  905  146 | 9.36 [5.86-14.96]  2.91 [1.66-5.08]  6.22 [4.49-8.61]  9.57 [4.47-20.49] | 36.2  9.3 |
| Blurred vision | Placebo  Sertraline  Amisulpride  Moclobemide  Reboxetine | 3 (Thase 96, Boyer 96B, Versiani 97)  1 (Thase 96)  1 (Boyer 96B)  1 (Versiani 97)  1 (Katona 99) | 629  170  146  211  129 | 5.21 [2.53-10.75]  2.95 [1.20-7.26]  3.50 [1.44-8.52]  2.22 [0.73-6.72]  1.65 [0.27-10.24] | 10.8 |
| Sweating | Placebo  Sertraline  Reboxetine | 2 (Thase 96, Versinai 97)  2 (Thase 96/ Rush/ Keller 98)  1 (Katona 99) | 483  905  129 | 8.21 [3.36-20.10]  2.64 [1.87-3.71]  1.58 [0.47-5.26] | 12.1  0 |
| Tremor | Placebo  Sertraline  Moclobemide | 2 (Thase 96, Versiani 97)  2 (Thase 96, Rush/ Keller 98)  1 (Versiani 97) | 483  905  211 | 11.97 [1.36-105.65]  3.19 [2.16-4.71]  3.31 [1.50-7.28] | 75.3  0 |
| Excessive thirst | Placebo  Amisulpride | 1 (Boyer 96B)  1 (Boyer 96 B) | 146  146 | 2.09 [1.01-4.34]  2.47 [1.17-5.25] |  |
| Paraesthesia | Placebo  Sertraline | 1 (Thase 96)  1 (Thase 96) | 276  270 | 2.13 [0.62-7.23]  1.33 [0.45-3.95] |  |
| Neurological events | Placebo  Amisulpride | 1 (Boyer 96B)  1 (Boyer 96B) | 146  146 | 4.37 [0.45-4.18]  1.37 [0.45-4.18] |  |
| ANS events | Minaprine | 1 (Salzmann 95) | 67 | 4.70 [1.61-13.78] |  |
| Endocrine events | Placebo | 1 (Boyer 96B) | 146 | 1.27 [0.08-21.12] |  |
| Sexual dysfunction | Placebo | 1 (Thase 96) | 276 | 2.52 [1.06-6.00] |  |
| Flushing | Placebo  Sertraline | 1 (Thase 96)  1 (Thase 96) | 276  270 | 11.03 [1.39-87.40]  10.55 [1.33-83.66] |  |
| Rash | Placebo  Sertraline | 1 (Thase 96)  1 (Thase 96) | 276  270 | 5.48 [1.18-25.48]  2.05 [0.68-6.16] |  |
| Hypotension | Placebo  Sertraline  Reboxetine | 1 (Thase 96)  1 (Thase 96)  1 (Katona 99) | 276  270  129 | 16.27 [0.92-287.83]  15.58 [0.55-275.59]  2.85 [0.53-15.27] |  |
| Dizziness | Placebo  Sertraline  Amisulpride  Moclobemide | 3 (Thase 96, Rush/ Keller 98, Versiani 97)  2 (Thase 96, Rush/ Keller 98)  1 (Boyer 96B)  1 (versiani 97) | 629  905  146  211 | 2.08 [1.41-3.09]  2.57 [1.86-3.55]  4.15 [1.84-9.38]  1.20 [0.60-2.39] | 0 |
| Palpitations | Placebo  Amisulpride  Sertraline  Reboxetine | 2 (Boyer 96B, Thase 96)  1 (Boyer 96B)  1 (Thase 96)  1 (Katona 99) | 422  146  270  129 | 3.04 [1.56-5.94]  2.94 [1.32-6.55]  2.06 [0.75-5.67]  1.08 [0.02-55.27] | 0 |
| **Amitryptiline (TCA)** |  |  |  |  |  |
| Constipation | Amisulpride | 1 (Ravizza 99) | 251 | 2.30 [1.00-5.30] |  |
| Gastrointestinal events | Amisulpride | 1 (Ravizza 99) | 251 | 1.31 [0.62-2.78] |  |
| Somnolence | Amisulpride | 1 (Ravizza 99) | 251 | 4.31 [1.95-9.50] |  |
| Fatigue | Amisulpride | 1 (Ravizza 99) | 251 | 3.01 [1.10-8.22] |  |
| Dry mouth | Amisulpride | 1 (Ravizza 99) | 251 | 5.62 [2.86-11.07] |  |
| Taste perversion | Amisulpride | 1 (Ravizza 99) | 251 | 8.03 [3.68-17.53] |  |
| Sweating | Amisulpride | 1 (Ravizza 99) | 251 | 10.87 [2.32-50.82] |  |
| Hot flushes | Amisulpride | 1 (Ravizza 99) | 251 | 7.31 [1.48-36.02] |  |
| Tremor | Amisulpride | 1 (Ravizza 99) | 251 | 22.61 [1.24-414.09] |  |
| Headache | Amisulpride | 1 (Ravizza 99) | 251 | 1.08 [0.35-3.34] |  |
| CNS events | Amisulpride | 1 (Ravizza 99) | 251 | 2.26 [1.29-3.97] |  |
| ANS events | Amisulpride | 1 (Ravizza 99) | 251 | 4.32 [2.38-7.86] |  |
| Neurological events | Amisulpride | 1 (Ravizza 99) | 251 | 12.46 [1.47-105.23] |  |
| Dizziness | Amisulpride | 1 (Ravizza 99) | 251 | 2.38 [0.77-7.32] |  |
| **Clomipramine (TCA)** |  |  |  |  |  |
| Constipation | Fluoxetine | 1 (Aguglia 95) | 48 | 3.13 [0.12-80.86] |  |
| Dry mouth | Fluoxetine | 1 (Aguglia 95) | 48 | 9.47 [1.06-84.37] |  |
| Diaphoresis | Fluoxetine | 1 (Aguglia 95) | 48 | 3.13 [0.12-80.86] |  |
| Hypotension | Fluoxetine | 1 (Aguglia 95) | 48 | 5.44 [0.25-119.92] |  |
| **Amisulpride (aPS)** |  |  |  |  |  |
| Weight gain | Fluoxetine  Amitryptiline  Lorazepam  Viloxazine  Acetyl-L-Carnitine | 2 (Bogetto 97, Smeraldi 98)  1 (Ravizza 99)  1 (Bogetto 97)  1 (Leon 94)  1 (Zanardi 06) | 304  251  25  78  204 | 3.50 [1.02-11.96]  1.27 [0.64-2.52]  14.29 [0.67-304.50]  4.34 [0.46-40.75]  2.14 [0.19-24.03] | 11.3 |
| Constipation | Fluoxetine | 1 (Smeraldi 98) | 278 | 2.69 [0.70-10.35] |  |
| Increased appetite | Fluoxetine | 1 (Smeraldi 98) | 278 | 9.00[0.48-168.79] |  |
| Abdominal pain | Fluoxetine | 1 (Smeraldi 98) | 278 | 1.97 [0.36-10.94] |  |
| Nausea/ Dyspepsia | Lorazepam | 1 (Bogetto 97) | 25 | 1.08 [0.02-59.02] |  |
| Somnolence/ Hypersomnia | Fluoxetine  Viloxazine (Somn)  Viloxazine (Hypers)  Acetyl-L-Carnitine | 2 (Bogetto 97, Smeraldi 98)  1 (Leon 94)  1 (Leon 94)  1 (Zanardi 06) | 304  78  78  204 | 2.14 [0.68-6.78]  1.65 [0.52-5.19]  2.63 [0.63-11.01]  5.41 [0.26-114.13] | 0 |
| Asthenia/ Fatigue | Fluoxetine  Acetyl-L-Carnitine | 2 (Bogetto 97, Smeraldi 98)  1 (Zanardi 06) | 304  204 | 2.17 [0.59-7.94]  1.06 [0.15-7.69] | 0 |
| Anxiety | Sertraline  Lorazepam | 1 (Bellino 97)  1 (Bogetto 97) | 49  25 | 3.53 [0.14-91.28]  3.52 [0.13-95.88] |  |
| Insomnia | Lorazepam | 1 (Bogetto 97) | 25 | 1.08 [0.02-59.02] |  |
| Dry mouth | Placebo | 1 (Boyer 96B) | 146 | 1.00 [0.45-2.23] |  |
| Blurred visison | Placebo | 1 (Boyer 96B) | 146 | 2.87 [0.73-11.29] |  |
| Neurological events | Placebo | 1 (Boyer 96B) | 146 | 3.18 [0.62-16.30] |  |
| Headache/ headheaviness | Fluoxetine  Viloxazine | 1 (Smeraldi 98)  1 (Leon 94) | 278  78 | 2.69 [0.70-10.35]  4.05 [0.78-20.89] |  |
| Tremor | Sertraline | 1 (Bellino 97) | 49 | 2.38 [0.20-28.14] |  |
| Hot flushes | Viloxazine | 1 (Leon 94) | 78 | 2.72 [0.49-14.96] |  |
| Endocrine events | Placebo  Amitryptiline  Sertraline  Imipramine | 1 (Boyer 96B)  1 (Ravizza 99)  1 (Amore 01)  1 (Boyer 96B) | 146  251  313  146 | 8.65 [1.01-73.75]  2.81 [1.12-7.06]  2.66 [1.18-5.98]  6.79 [0.79-58.29] |  |
| Galactorrhea/ Lactation | Viloxazine  Amitryptiline  Fluoxetine  Acetyl-L-Carnitine | 1 (Leon 94)  1 (Ravizza 99)  1 (Smeraldi 98)  1 (Zanardi 06) | 78  251  278  204 | 9.25 [1.92-44.50]  16.37 [0.96-277-84]  7.99 [0.42-150.64]  14.68 [0.82-263.95] |  |
| Libido reduction | Fluoxetine  Lorazepam | 2 (Bogetto 97, Smeraldi 98)  1 (Bogetto 97) | 304  25 | 3.88 [1.16-12.98]  6.00 [0.56-63.99] | 0 |
| Increased prolactin | Acetyl-L-Carnitine | 1 (Zanardi 06) | 204 | 18.57 [2.40-143.48] |  |
| Amenorrhea | Amitryptiline  Fluoxetine  Lorazepam | 1 (Ravizza 99)  2 (Bogetto 97, Smeraldi 98)  1 (Bogetto 97) | 251  304  25 | 7.33 [0.93-57.61]  8.37 [0.99-70.68]  6.43 [0.28-150.23] | 0 |
| Breast pain | Amitryptiline | 1 (Ravizza 99) | 251 | 3.25 [0.71-14.89] |  |
| Micturition difficulties | Placebo | 1 (Boyer 96B) | 146 | 3.04 [0.12-75.91] |  |
| Dizziness | Fluoxetine | 2 (Bogetto 97, Smeraldi 98) | 304 | 1.42 [0.44-4.59] | 0 |
| Edema | Acetyl-L-Carnitine | 1 (Zanardi 06) | 204 | 5.41 [0.26-114-13] |  |
| **Flupenthixol (aPS)** |  |  |  |  |  |
| Weight gain | Ritanserin | 1 (Geisler 92) | 69 | 1.17 [0.29-4.78] |  |
| Dyspepsia | Ritanserin | 1 (Geisler 92) | 69 | 2.83[0.11-72.02] |  |
| Nausea/ Vomiting | Ritanserin | 1 (Geisler 92) | 69 | 1.88 [0.16-21.78] |  |
| Depression | Ritanserin | 1 (Geisler 92) | 69 | 4.85 [0.22-105.06] |  |
| Reduced duration of sleep | Ritanserin | 1 (Geisler 92) | 69 | 7.00 [0.35-141.00] |  |
| Lightheadedness | Ritanserin | 1 (Geisler 92) | 69 | 2.83 [0.11-72.02] |  |
| Increased salivation | Ritanserin | 1 (Geisler 92) | 69 | 2.83 [0.11-72.02] |  |
| Increased sweating | Ritanserin | 1 (Geisler 92) | 69 | 1.88 [0.16-21.78] |  |
| Dystonia | Ritanserin | 1 (Geisler 92) | 69 | 4.85 [0.22-105.06] |  |
| Accommodation disturbances | Ritanserin | 1 (Geisler 92) | 69 | 2.00 [0.46-8.75] |  |
| Amenorrhea | Ritanserin | 1 (Geisler 92) | 69 | 2.83 [0.11-72.02] |  |
| Polyuria | Ritanserin | 1 (Geisler 92) | 69 | 1.88 [0.16-21.78] |  |
| **Duloxetine (SNRI)** |  |  |  |  |  |
| Decreased appetite | Placebo | 1 (Hellerstein 12) | 65 | 2.29 [0.60-8.69] |  |
| Nausea | Placebo | 1 (Hellerstein 12) | 65 | 1.20 [0.32-4.49] |  |
| Constipation | Placebo | 1 (Hellerstein 12) | 65 | 2.71 [0.48-15.29] |  |
| Agitation | Placebo | 1 (Hellerstein 12) | 65 | 5.85 [1.14-30.14] |  |
| Fatigue | Placebo | 1 (Hellerstein 12) | 65 | 1.13 [0.36-3.50] |  |
| Vivid dreams | Placebo | 1 (Hellerstein 12) | 65 | 8.59 [0.98-75.21] |  |
| Decreased sleep | Placebo | 1 (Hellerstein 12) | 65 | 1.57 [0.39-6.27] |  |
| Anxiety | Placebo | 1 (Hellerstein 12) | 65 | 1.50 [0.23-9.73] |  |
| Headache | Placebo | 1 (Hellerstein 12) | 65 | 2.65 [0.61-11.52] |  |
| Dry mouth | Placebo | 1 (Hellerstein 12) | 65 | 5.63 [0.61-51.60] |  |
| Delayed orgasm | Placebo | 1 (Hellerstein 12) | 65 | 3.12 [0.30-31.90] |  |
| Rash | Placebo | 1 (Hellerstein 12) | 65 | 3.12 [0.30-31.90] |  |
| **Reboxetine (SNRI)** |  |  |  |  |  |
| Insomnia | Imipramine | 1 (Katona 99) | 129 | 8.86 [0.47-168.05] |  |
| Agitation | Imipramine | 1 (Katona 99) | 129 | 2.86 [0.29-28.24] |  |
| Dry mouth | Imipramine | 1 (Katona 99) | 129 | 1.57 [0.71-3.44] |  |
| Headache/ migraine | Imipramine | 1 (Katona 99) | 129 | 1.41 [0.23-8.71] |  |
| Tremor | Imipramine | 1 (Katona 99) | 129 | 3.87 [0.42-35.64] |  |
| Confusion | Imipramine | 1 (Katona 99) | 129 | 2.86 [0.29-28.24] |  |
| Paraesthesia | Imipramine | 1 (Katona 99) | 129 | 2.86 [0.29-28.24] |  |
| **Viloxazine (SNRI)** |  |  |  |  |  |
| Constipation | Amisulpride | 1 (Leon 94) | 78 | 1.59 [0.41-6.15] |  |
| Insomnia | Amisulpride | 1 (Leon 94) | 78 | 4.34 [0.46-40.75] |  |
| Insufficient sleep | Amisulpride | 1 (Leon 94) | 78 | 5.59 [0.62-50.25] |  |
| Dry mouth | Amisulpride | 1 (Leon 94) | 78 | 2.26 [0.62-8.24] |  |
| Bitter taste | Amisulpride | 1 (Leon 94) | 78 | 4.05 [0.78-20.89] |  |
| Amenorrhea | Amisulpride | 1 (Leon 94) | 78 | 1.29 [0.32-5.20] |  |
| Dizziness | Amisulpride | 1 (Leon 94) | 78 | 2.72 [0.49-14.96] |  |
| **Sertraline (SSRI)** |  |  |  |  |  |
| Palpitation | Placebo | 1 (Thase 96) | 274 | 2.14 [0.52-8.74] |  |
| Constipation | Placebo | 2 (Thase 96, Ravindran 00) | 584 | 2.06 [1.11-3.82] |  |
| Diarrhea | Placebo  Imipramine | 2 (Thase 96, Ravindran 00)  2 (Thase 96, Rush/ Keller 98) | 584  805 | 2.13 [1.27-3.57]  4.48 [2.79-7.19] | 0  0 |
| Dyspepsia | Placebo  Amisulpride | 2 (Thase 96, Ravindran 00)  1 (Bellino 97) | 584  49 | 2.02 [1.20-3.42]  9.40 [0.48-185.25] | 0 |
| Nausea | Placebo  Imipramine  Amisulpride | 2 (Thase 96, Ravindran 00)  2 (Thase 96, Rush/ Keller 98)  1 (Bellino 97) | 584  805  49 | 1.37 [0.92-2.04]  1.28 [0.94-1.74]  9.40 [0.48-185.25] | 0  0 |
| Vomiting | Placebo | 2 (Thase 96, Ravindran 00) | 584 | 1.86 [0.77-4.51] | 0 |
| Flatulence | Placebo | 1 (Ravindran 00) | 310 | 2.93 [1.12-7.66] |  |
| Abdominal pain | Placebo | 1 (Ravindran 00) | 310 | 2.44 [1.08-5.50] |  |
| Gastrointestinal symptoms | Amisulpride | 1 (Amore 01) | 313 | 1.70 [0.97-2.98] |  |
| Insomnia | Placebo  Imipramine  Amisulpride | 2 (Thase 96, Ravindran 00)  2 (Thase 96, Rush/ Keller 98)  1 (Bellino 97) | 584  805  49 | 1.51 [1.00-2.27]  1.90 [1.34-2.70]  2.76 [0.11-71.40] | 0  0 |
| Somnolence | Placebo | 2 (Thase 96, Ravindran 00) | 584 | 1.95 [1.18-3.21] |  |
| Anorexia | Placebo  Imipramine | 1 (Thase 96)  1 (Thase 96) | 274  270 | 4.61 [1.50-14.17]  1.09 [0.52-2.31] |  |
| Agitation | Placebo  Imipramine | 1 (Thase 96)  2 (Thase 96) | 274  270 | 4.91 [1.37-17.63]  1.22 [0.53-2.83] |  |
| Fatigue | Placebo | 1 (Ravindran 00) | 310 | 2.77 [0.86-8.89] |  |
| Anxiety | Placebo | 2 (Thase 96, Ravindran 00) | 584 | 1.81 [0.89-3.69] | 0 |
| Dry mouth | Placebo | 2 (Thase 96, Ravindran 00) | 584 | 1.71 [1.06-2.75] | 0 |
| Sweating | Placebo | 2 (Thase 96, Ravindran 00) | 584 | 3.94 [1.13-13.67] | 63.5 |
| Tremor | Placebo | 2 (Thase 96, Ravindran 00) | 584 | 19.96 [4.75-83.86] | 0 |
| Abnormal vision | Placebo | 1 (Thase 96) | 274 | 1.87 [0.54-6.55] |  |
| Paraesthesia | Placebo | 1 (Thase 96) | 274 | 1.59 [0.44-5.78] |  |
| Headache | Imipramine  Amisulpride | 2 (Thase 96, Rush/ Keller 98)  1 (Bellino 97) | 805  49 | 1.27 [0.96-1.69]  2.76 [0.11-71.40] | 0 |
| Sexual dysfunction | Placebo  Imipramine | 1 (Thase 96)  2 (Thase 96, Rush/ Keller 98) | 274  805 | 2.73 [1.15-6.46]  1.12 [0.74-1.68] | 0 |
| Influenza like symptoms | Placebo | 1 (Ravindran 00) | 310 | 1.09 [0.41-2.90] |  |
| Pharyngitis | Placebo | 1 (Ravindran 00) | 310 | 1.30 [0.44-3.83] |  |
| Upper respiratory tract infection | Placebo | 1 (Ravindran 00) | 310 | 1.40 [0.52-3.77] |  |
| Micturition disorder | Placebo | 1 (Thase 96) | 274 | 1.05 [0.06-16.88] |  |
| Rash | Placebo | 1 (Thase 96) | 274 | 2.67 [0.51-14.03] |  |
| Hypotension | Placebo | 1 (Thase 96) | 274 | 1.04 [0.02-53.03] |  |
| Dizziness | Placebo | 1 (Thase 96, Ravindran 00) | 584 | 1.70 [0.44-6.55] | 81.9 |
| **Paroxetine (SSRI)** |  |  |  |  |  |
| Nausea | Placebo | 1 (Ravindran 2013) | 40 | 1.67 [0.34-7.18] |  |
| Diarrhea | Placebo | 1 (Ravindran 2013) | 40 | 1.42 [0.21-9.55] |  |
| Fatigue | Placebo | 1 (Ravindran 2013) | 40 | 7.38 [0.35-153.40] |  |
| Headache | Placebo | 1 (Ravindran 2013) | 40 | 1.88 [0.45-8.18] |  |
| Sweating | Placebo | 1 (Ravindran 2013) | 40 | 10.03 [0.50-200.67] |  |
| Sexual dysfunction | Placebo | 1 (Ravindran 2013) | 40 | 5.63 [0.59-53.38] |  |
| **Fluoxetine (SSRI)** |  |  |  |  |  |
| Tachycardia | Acetyl-l-Carnitine | 1 (Bersani 2013) | 80 | 3.23 [0.13-81.86] |  |
| Nausea/ Vomiting | Amisulpride  Acetyl-l-Carnitine | 1 (Smeraldi 98)  1 (Bersani 2013) | 278  80 | 13.44 [1.72-104.84]  2.03 [0.65-8.67] |  |
| Nausea | Clomipramine | 1 (Aguglia 95) | 48 | 1.84 [0.39-8.77] |  |
| Dyspepsia | Amisulpride | 1 (Smeraldi 98) | 278 | 2.63 [0.05-13.80] |  |
| Nausea/ Dyspepsia | Amisulpride  Lorazepam | 1 (Bogetto 97)  1 (Bogetto 97) | 26  27 | 10.71 [0.51-225.23]  11.57 [0.55-242.18] |  |
| Diarrhea | Acetyl-l-carnitine | 1 (Bersani 2013) | 80 | 2.23 [0.38-12.92] |  |
| Anorexia | Amisulpride  Clomipramine  Acetyl-l-carnitine | 1 (Smeraldi 98)  1 (Aguglia 95)  1 (Bersani 2013) | 276  48  80 | 4.89 [1.04-23.04]  3.13 [0.12-80.86]  7.27 [0.83-63.48] |  |
| Nervousness/ Shakiness/ Anxiety | Placebo  Amisulpride  Lorazepam | 1 (Hellerstein 93)  1 (Bogetto 97)  1 (Bogetto 97) | 35  26  27 | 2.00 [0.41-9.76]  3.00 [0.27-33.49]  8.22 [0.38-177.86] |  |
| Insomnia | Amisulpride  Lorazepam  Acetyl-l-carnitine | 2 (Bogetto 97, Smerladi 98)  1 (Bogetto 97)  1 (Bersani 2013) | 304  27  80 | 1.63 [0.38-6.95]  8.21 [0.38-177.86]  1.63 [0.26-10.29] | 26.0 |
| Agitation | Amsulpride  Acetyl-l-carnitine | 1 (Smeraldi 98)  1 (Bersani 2013) | 278  80 | 3.13 [0.32-30.51]  5.53 [0.26-119.10] |  |
| Increase in motor activities | Acetyl-l-carnitine | 1 (Bersani 2013) | 80 | 4.27 [0.83-21.98] |  |
| Sweating | Placebo  Amisulpride  Acetyl-l-carnitine | 1 (Hellerstein 93)  1 (Smeraldi 98)  1 (Bersani 2013) | 35  278  80 | 2.81 [0.26-30.09]  3.13 [0.32-30.51]  5.53 [0.26-119.10] |  |
| Headache | Clomipramine  Acetyl-l-carnitine | 1 (Aguglia 95)  1 (Bersani 2013) | 48  80 | 5.44 [0.25-119.92]  1.44 [0.30-6.93] |  |
| Dry mouth | Acetyl-l-carnitine | 1 (Bersani 2013) | 80 | 1.05 [0.06-17.44] |  |
| Abnormal accommodation | Amisulpride  Acetyl-l-carnitine | 1 (Smeraldi 98)  1 (Bersani 2013) | 278  80 | 3.13 [0.32-30.51]  5.53 [0.26-119.10] |  |
| Neurological symptoms | Amisulpride | 1 (Smeraldi 98) | 278 | 1.03 [0.02-5.19] |  |
| Tremor | Acetyl-l-carnitine | 1 (Bersani 2013) | 80 | 2.40 [0.66-8.69] |  |
| Akathisia | Acetyl-l-carnitine | 1 (Bersani 2013) | 80 | 3.2 [0.13-81.86] |  |
| Sexual dysfunction | Placebo | 1 (Hellerstein 93) | 35 | 2.81 [0.26-30.10] |  |
| Libido reduction | Lorazepam | 1 (Bogetto 97) | 27 | 2.00 [0.16-25.12] |  |
| Muscle/ joint pain | Placebo | 1 (Hellerstein 93) | 35 | 9.58 [0.47-194.03] |  |
| Hypotension | Acetyl-lcarnitine | 1 (Bersani 2013) | 80 | 3.23 [0.12-81.86] |  |
| Dizziness | Acetyl-l-carnitine | 1 (Bersani 2013) | 80 | 1.44 [0.30-6.93] |  |
| **Escitalopram (SSRI)** |  |  |  |  |  |
| Decreased libido | Placebo | 1 (Hellerstein 10) | 32 | 7.64 [0.80-73.15] |  |
| **Moclobemide (MAOI)** |  |  |  |  |  |
| Nausea | Placebo  Imipramine | 1 (Versiani 97)  1 (Versiani 97) | 212  211 | 2.01 [0.88-4.55]  1.25 [0.60-2.62] |  |
| Insomnia | Placebo  Imipramine | 1 (Versiani 97)  1 (Versiani 97) | 212  211 | 1.64 [0.75-3.57]  1.79 [0.80-3.96] |  |
| Anxiety | Fluoxetine | 1 (Duarte 96) | 42 | 3.15 [0.12-81.97] |  |
| Dry mouth | Placebo | 1 (Versiani 97) | 212 | 2.15 [1.15-3.99] |  |
| Tremor | Placebo | 1 (Versiani 97) | 212 | 1.41 [0.52-3.87] |  |
| Sweating | Placebo | 1 (Versiani 97) | 212 | 5.00 [0.57-43.55] |  |
| Blurred vision | Placebo | 1 (Versiani 97) | 212 | 1.21 [0.32-4.65] |  |
| Headache | Placebo  Imipramine | 1 (Versiani 97)  1 (Versiani 97) | 212  211 | 1.85 [0.86-3.99]  1.31 [0.64-2.68] |  |
| Dizziness | Placebo | 1 (Versiani 97) | 212 | 2.01 [0.88-4.55] |  |
| **Lorazepam (Benz)** |  |  |  |  |  |
| Weight gain | Fluoxetine | 1 (Bogetto 97) | 17 | 1.07 [0.02-58.34] |  |
| Somnolence | Fluoxetine  Amisulpride | 1 (Bogetto 97)  1 (Bogetto 97) | 17  15 | 9.67 [0.45-209.76]  8.33 [0.38-182.29] |  |
| Asthenia | Fluoxetine  Amisulpride | 1 (Bogetto 97)  1 (Bogetto 97) | 17  15 | 13.74 [0.65-288.78]  11.84 [0.56-250.89] |  |
| Amenorrhea | Fluoxetine | 1 (Bogetto 97) | 17 | 1.07 [0.02-58.34] |  |
| Dizziness | Fluoxetine  Amisulpride | 1 (Bogetto 97)  1 (Bogetto 97) | 17  15 | 2.36 [0.19-29.71]  5.43 [0.23-126.77] |  |
| **Ritanserin (oAD)** |  |  |  |  |  |
| Palpitations | Flupenthixol | 1 (Geisler 92) | 69 | 2.26 [0.20-26.13] |  |
| Changes in weight | Flupenthixol | 1 (Geisler 92) | 69 | 1.09 [0.02-56.52] |  |
| Weight loss | Flupenthixol | 1 (Geisler 92) | 69 | 3.37 [0.13-85.72] |  |
| Diarrhea | Flupenthixol | 1 (Geisler 92) | 69 | 2.34 [0.40-13.74] |  |
| Borborygmi | Flupenthixol | 1 (Geisler 92) | 69 | 5.81 [0.27-125.40] |  |
| Tension | Flupenthixol | 1 (Geisler 92) | 69 | 1.96 [0.43-8.96] |  |
| Emotional indifference | Flupenthixol | 1 (Geisler 92) | 69 | 5.81 [0.27-125.40] |  |
| Increased dream activity | Flupenthixol | 1 (Geisler 92) | 69 | 1.10 [0.21-5.87] |  |
| Increased duration of sleep | Flupenthixol | 1 (Geisler 92) | 69 | 1.10 [0.21-5.87] |  |
| Sleepiness | Flupenthixol | 1 (Geisler 92) | 69 | 3.78 [0.71-20.23] |  |
| Failing memory | Flupenthixol | 1 (Geisler 92) | 69 | 1.10 [0.15-8.26] |  |
| Concentration difficulties | Flupenthixol | 1 (Geisler 92) | 69 | 1.11 [0.29-4.23] |  |
| Asthenia | Flupenthixol | 1 (Geisler 92) | 69 | 1.10 [0.25-4.82] |  |
| Psychic events | Flupenthixol | 1 (Geisler 92) | 69 | 1.12 [0.37-3.43] |  |
| Reduced salivation | Flupenthixol | 1 (Geisler 92) | 69 | 1.78 [0.45-6.96] |  |
| Hyperkinesia | Flupenthixol | 1 (Geisler 92) | 69 | 3.38 [0.13-85.72] |  |
| Headache | Flupenthixol | 1 (Geisler 92) | 69 | 3.50 [0.35-35.44] |  |
| Rigidity | Flupenthixol | 1 (Geisler 92) | 69 | 1.09 [0.07-18.22] |  |
| Tremor | Flupenthixol | 1 (Geisler 92) | 69 | 3.50 [0.35-35.44] |  |
| Neurological events | Flupenthixol | 1 (Geisler 92) | 69 | 1.10 [0.21-5.87] |  |
| Autonomic events | Flupenthixol | 1 (Geisler 92) | 69 | 1.33 [0.42-4.17] |  |
| Increased sexual desire | Flupenthixol | 1 (Geisler 92) | 69 | 3.38 [0.13-85.72] |  |
| Micturition disturbances | Flupenthixol | 1 (Geisler 92) | 69 | 3.37 [0.13-85.72] |  |
| Pruritus | Flupenthixol | 1 (Geisler 92) | 69 | 1.10 [0.15-8.26] |  |
| Rash | Flupenthixol | 1 (Geisler 92) | 69 | 3.37 [0.13-85.72] |  |
| Dizziness | Flupenthixol | 1 (Geisler 92) | 69 | 4.58 [0.88-23.89] |  |
| Other events | Flupenthixol | 1 (Geisler 92) | 69 | 1.67 [0.47-5.88] |  |
| **Acetyl-L-Carnitine (cmpl)** |  |  |  |  |  |
| Dyspepsia/ Dysphagia | Amisulpride | 1 (Zanardi 06) | 204 | 2.88 [0.29-28.18] |  |
| Constipation | Fluoxetine | 1 (Bersani 13) | 80 | 3.00 [0.30-30.15] |  |
| Depression | Fluoxetine | 1 (Bersani 13) | 80 | 5.00 [0.23-107.61] |  |
| Sleepiness | Fluoxetine | 1 (Bersani 13) | 80 | 1.95 [0.17-22.40] |  |
| Reduction in motor activities | Fluoxetine | 1 (Bersani 13) | 80 | 2.93 [0.12-74.06] |  |
| Nasal congestion | Fluoxetine | 1 (Bersani 13) | 80 | 1.95 [0.17-22.40] |  |
| Hypertension | Fluoxetine | 1 (Bersani 13) | 80 | 2.93 [0.12-74.06] |  |
| Dermatologic events | Fluoxetine | 1 (Bersani 13) | 80 | 2.93 [0.12-74.06] |  |
| **Placebo** |  |  |  |  |  |
| Headache | Sertraline  Imipramine | 2 (Thase 96, Ravindran 00)  2 (Thase 96, Versiani 97) | 584  483 | 1.18 [0.84-1.67]  1.05 [0.59-1.89] | 0  40.8 |
| Increased appetite | Sertraline | 1 (Thase 96) | 274 | 1.61 [0.38-7.12] |  |
| Back pain | Sertraline | 1 (Ravindran 00) | 310 | 1.18 [0.44-3.13] |  |
| Diarrhea | Imipramine | 1 (Thase 96) | 276 | 1.41 [0.60-3.23] |  |
| Insomnia | Imipramine | 1 (Thase 96) | 276 | 1.35 [0.79-2.33] |  |
| Excessive thirst | Amisulpride | 1 (Boyer 96b) | 146 | 1.18 [0.53-2.63] |  |
| Constipation | Amisulpride  Moclobemide | 1 (Boyer 96b)  1 (Versiani 97) | 146  212 | 1.35 [0.65-2.94]  1.49 [0.65-3.45] |  |
| Dizziness | Amisulpride  Duloxetine | 1 (Boyer 96b)  1 (Hellerstein 12) | 146  57 | 2.22 [0.95-5.26]  2.27 [0.38-14.29] |  |
| Palpitation | Amisulpride  Duloxetine | 1 (Boyer 96b)  1 (Hellerstein 12) | 146  57 | 1.11 [0.45-2.70]  3.33 [0.33-33.33] |  |
| Gastrointestinal upset | Duloxetine  Escitalopram | 1 (Hellerstein 12)  1 (Hellerstein 10) | 57  32 | 1.49 [0.47-4.67]  10 [0.46-100] |  |
| Decreased libido | Duloxetine | 1 (Hellerstein 12) | 57 | 3.33 [0.33-33.33] |  |
| Decreased concentration | Duloxetine | 1 (Hellerstein 12) | 57 | 3.33 [0.33-33.33] |  |
| Sexual events | Duloxetine | 1 (Hellerstein 12) | 57 | 1.04 [0.14-7.69] |  |

OR=odds ratio; CI=confidence interval.

Comparisons are listed under the agents (headings on the left), where the respective adverse events were more frequent than for the indicated comparator (all odds ratios are higher than 1).
