# Supplementary material for: Comparative Safety of Pharmacologic Treatments for Persistent Depressive Disorder: A Systematic Review and Network Meta-Analysis
Source: PLoS One. 2016 May 17;11(5):e0153380. doi: 10.1371/journal.pone.0153380 (PMC4871495; doi:10.1371/journal.pone.0153380)
Supplement: S6 Table — (DOCX) [file pone.0153380.s010.docx]

**S6 Table. Absolute numbers of patients experiencing specific adverse events**

| **Study ID**  **Adverse Event** | **Absolute numbers of patients experiencing specific adverse events** | | |
| --- | --- | --- | --- |
| **Aguglia (1995)** | **Fluoxetine (n=24)** | **Clomipramine (n=24)** |  |
| Nausea | 5 | 3 |  |
| Dry mouth | 1 | 7 |  |
| Headache | 2 | 0 |  |
| Anorexia | 1 | 0 |  |
| Orthostatic hypotension | 0 | 2 |  |
| Constipation | 0 | 1 |  |
| Diaphoresis | 0 | 1 |  |
| **Amore (2001)** | **Amisulpride (n=157)** | **Sertraline (n=156)** |  |
| Gastrointestinal events | 25 | 38 |  |
| Endocrine events | 22 | 9 |  |
| **Bellino (1997)** | **Amisulpride (n=23)** | **Sertraline (n=26)** |  |
| Nausea | 0 | 4 |  |
| Dyspepsia | 0 | 4 |  |
| Insomnia | 0 | 1 |  |
| Headache | 0 | 1 |  |
| Tremor | 2 | 1 |  |
| Anxiety | 1 | 0 |  |
| **Bersani (2013)** | **Acetyl-L-Carnitine (n=41)** | **Fluoxetine (n=39)** |  |
| Tremor | 4 | 8 |  |
| Nausea/vomiting | 4 | 7 |  |
| Increase in motor activity | 2 | 7 |  |
| Anorexia/ loss of appetite | 1 | 6 |  |
| Headache | 3 | 4 |  |
| Diarrhea | 2 | 4 |  |
| Dizziness | 3 | 4 |  |
| Insomnia | 2 | 3 |  |
| Agitation | 0 | 2 |  |
| Sweating | 0 | 2 |  |
| Cloudy vista | 0 | 2 |  |
| Hypotension | 0 | 1 |  |
| Constipation | 3 | 1 |  |
| Dry mouth | 1 | 1 |  |
| Tachycardia | 0 | 1 |  |
| Akathisia | 0 | 1 |  |
| Nasal congestion | 2 | 1 |  |
| Depression | 2 | 0 |  |
| Sleepiness | 2 | 1 |  |
| Reduction in motor activity | 1 | 0 |  |
| Hypertension | 1 | 0 |  |
| Dermatologic symptoms | 1 | 0 |  |
| **Bogetto (1997)** | **Amisulpride (n=12)** | **Fluoxetine (n=14)** | **Lorazepam (n=13)** |
| Nausea/Dyspepsia | 0 | 4 | 0 |
| Insomnia | 0 | 3 | 0 |
| Anxiety | 1 | 3 | 0 |
| Libido Reduction | 4 | 2 | 1 |
| Amenorrhea | 2 | 0 | 0 |
| weight gain | 4 | 0 | 0 |
| Somnolence | 0 | 0 | 3 |
| Dizziness | 0 | 1 | 2 |
| Asthenia | 0 | 0 | 4 |
| **Boyer (1996A)** | **Placebo (n=108)** | **Amisulpride (n=104)** | **Amineptine (n=111)** |
| Galactorrhea |  | 8 (out of 73 female) |  |
| **Duarte (1996)** | **Moclobemide (n=21)** | **Fluoxetine (n=21)** |  |
| Anxiety | 1 | 0 |  |
| **Geisler (1992)** | **Ritanserine (n=33)** | **Fluenthixol (n=36)** |  |
| Psychic Symptoms | 8 | 8 |  |
| concentration difficulties | 5 | 5 |  |
| Asthenia | 4 | 4 |  |
| Sleepiness | 6 | 2 |  |
| Failing memory | 2 | 2 |  |
| Depression | 0 | 2 |  |
| Tension | 5 | 3 |  |
| Increased duration of sleep | 3 | 3 |  |
| Reduced duration of sleep | 0 | 3 |  |
| Increased dream activity | 3 | 3 |  |
| Emotional indifference | 2 | 0 |  |
| Lightheadedness | 0 | 1 |  |
| Neurological Symptoms | 3 | 3 |  |
| Dystonia | 0 | 2 |  |
| Rigidity | 1 | 1 |  |
| Hyperkinesia | 1 | 0 |  |
| Tremor | 3 | 1 |  |
| Autonomic Symptoms | 8 | 7 |  |
| Accommodation disturbances | 3 | 6 |  |
| Increased salivation | 0 | 1 |  |
| Reduced salivation | 6 | 4 |  |
| Nausea/Vomiting | 1 | 2 |  |
| Diarrhea | 4 | 2 |  |
| Polyuria | 1 | 2 |  |
| Orthostatic Dizziness | 7 | 2 |  |
| Palpitations | 2 | 1 |  |
| Increased sweating | 1 | 2 |  |
| Micturition disturbances | 1 | 0 |  |
| Dyspepsia | 0 | 1 |  |
| Borborygmi | 2 | 0 |  |
| Other Symptoms | 7 | 5 |  |
| Rash | 1 | 0 |  |
| Pruritus | 2 | 2 |  |
| Weight gain | 4 | 5 |  |
| Weight loss | 1 | 0 |  |
| Amenorrhea | 0 | 1 |  |
| Increased sexual desire | 1 | 0 |  |
| Headache | 3 | 1 |  |
| **Hellerstein (1993)** | **Fluoxetine (n=19)** | **Placebo (n=16)** |  |
| Nervousness/Shakiness | 6 | 3 |  |
| Muscle/joint pain | 4 | 0 |  |
| Sexual dysfunction | 3 | 1 |  |
| Sweating | 3 | 1 |  |
| **Hellerstein (2010)** | **Escitalopram (n=17)** | **Placebo (n=15)** |  |
| Upset stomach | 0 | 3 |  |
| Decreased libido | 6 | 1 |  |
| **Hellerstein (2012)** | **Duloxetine (n=29)** | **Placebo (n=28)** |  |
| Agitation | 9 | 2 |  |
| Fatigue | 9 | 8 |  |
| Decreased appetite | 8 | 4 |  |
| Gastrointestinal upset | 7 | 9 |  |
| Headache | 7 | 3 |  |
| Vivid Dreams | 7 | 1 |  |
| Decreased sleep | 6 | 4 |  |
| Nausea | 6 | 5 |  |
| Constipation | 5 | 2 |  |
| Dry Mouth | 5 | 1 |  |
| Anxiety | 3 | 2 |  |
| Delayed orgasm | 3 | 1 |  |
| Rash | 3 | 1 |  |
| Dizziness | 2 | 4 |  |
| Sexual side effects | 2 | 2 |  |
| Decreased concentration | 1 | 3 |  |
| Decreased libido | 1 | 3 |  |
| Palpitations | 1 | 3 |  |
| **Katona (1999)** | **Reboxetine (n=67)** | **Imipramine (n=62)** |  |
| Nausea | 9 | 9 |  |
| Constipation | 9 | 11 |  |
| Dry mouth | 21 | 14 |  |
| Increased sweating | 5 | 7 |  |
| Insomnia | 4 | 0 |  |
| Agitation(anxiety/nervousness | 3 | 1 |  |
| Somnolence | 0 | 3 |  |
| Hypotension | 2 | 5 |  |
| Tachycardia | 0 | 0 |  |
| Headache/migraine | 3 | 2 |  |
| Tremor | 4 | 1 |  |
| Confusion | 3 | 1 |  |
| Paranesthesia | 3 | 1 |  |
| Blurred vison | 2 | 3 |  |
| Asthenia/Fatigue | 1 | 3 |  |
| Hesitancy | 1 | 5 |  |
| Infection | 1 | 3 |  |
| **Leon (1994)** | **Amisulpride (n=39)** | **Viloxacine (n=39)** |  |
| Galactorrhea | 13 | 2 |  |
| Somnolence | 9 | 6 |  |
| Hypersomnia | 7 | 3 |  |
| Head Heaviness | 7 | 2 |  |
| Hot flashes | 5 | 2 |  |
| Amenorrhea | 4 | 5 |  |
| Weight gain | 4 | 1 |  |
| Dry Mouth | 4 | 8 |  |
| Constipation | 4 | 6 |  |
| Bitter Taste | 2 | 7 |  |
| Dizziness | 2 | 5 |  |
| Insomnia | 1 | 4 |  |
| Insufficient sleep | 1 | 5 |  |
| **Ravindran (2000)** | **Sertraline (n=158)** | **Placebo (n=152)** |  |
| Dry Mouth | 17 | 10 |  |
| Increased sweating | 22 | 3 |  |
| Dizziness | 20 | 6 |  |
| Headache | 48 | 51 |  |
| Tremor | 22 | 1 |  |
| Abdominal pain | 21 | 9 |  |
| Constipation | 10 | 5 |  |
| Diarrhea | 20 | 11 |  |
| Dyspepsia | 28 | 15 |  |
| Flatulence | 17 | 6 |  |
| Nausea | 33 | 27 |  |
| Vomiting | 9 | 6 |  |
| Back pain | 8 | 9 |  |
| Fatigue | 11 | 4 |  |
| Influenza like symptoms | 9 | 8 |  |
| Anxiety | 13 | 5 |  |
| Insomnia | 35 | 25 |  |
| Somnolence | 18 | 11 |  |
| Ejaculation disorder | 5 (out of 54 male) | 0 |  |
| Pharyngitis | 8 | 6 |  |
| Upper respiratory tract infection | 10 | 7 |  |
| **Ravindran (2001)** | **Paroxetine (n=21)** | **Placebo (n=19)** |  |
| Headache | 7 | 4 |  |
| Nausea | 5 | 3 |  |
| Sexual dysfunction | 5 | 1 |  |
| Diarrhea | 3 | 2 |  |
| Sweating | 4 | 0 |  |
| Fatigue | 3 | 0 |  |
| **Ravizza (1999)** | **Amisulpride (n=165)** | **Amitriptyline (n=85)** |  |
| CNS events | 39 | 35 |  |
| ANS events | 26 | 38 |  |
| Gastrointestinal events | 20 | 13 |  |
| Endocrine events | 29 | 6 |  |
| Dry mouth | 16 | 32 |  |
| Taste perversion | 10 | 29 |  |
| Somnolence | 11 | 20 |  |
| Weight gain | 33 | 14 |  |
| Constipation | 12 | 13 |  |
| Amenorrhea | 13 | 1 |  |
| Fatigue | 7 | 10 |  |
| Sweating increased | 2 | 10 |  |
| Lactation | 14 | 0 |  |
| Dizziness | 6 | 7 |  |
| Hot flushes | 2 | 7 |  |
| Breast pain | 12 | 2 |  |
| Headache | 9 | 5 |  |
| Tremor | 0 | 5 |  |
| Weight gain >=5% | 30 | 9 |  |
| Neurological events | 1 | 6 |  |
| **Rush (1998) / Keller (1998)** | **Sertraline (n=426)** | **Imipramine (n=209)** |  |
| Nausea | 131 | 51 |  |
| Diarrhea | 112 | 13 |  |
| Dyspepsia | 67 | 41 |  |
| Constipation | 47 | 71 |  |
| Insomnia | 115 | 37 |  |
| Somnolence | 88 | 58 |  |
| Nervousness | 42 | 27 |  |
| Headache | 168 | 67 |  |
| Dizziness | 74 | 75 |  |
| Tremor | 34 | 48 |  |
| Dry mouth | 148 | 156 |  |
| Sweating | 60 | 61 |  |
| Fatigue | 52 | 28 |  |
| Micturition disorder | 9 | 25 |  |
| Sexual dysfunction | 57 | 25 |  |
| **Salzmann (1995)** | **Minaprine (n=33)** | **Imipramine (n=34)** |  |
| ANS events | 7 | 19 |  |
| **Smeraldi (1998)** | **Amisulpride (n=141)** | **Fluoxetine (n=137)** |  |
| Weight gain | 13 | 5 |  |
| Nausea, Vomiting | 1 | 12 |  |
| Insomnia | 10 | 11 |  |
| Dry Mouth | 10 | 10 |  |
| Anorexia | 2 | 9 |  |
| Somnolence | 9 | 4 |  |
| Loss of Libido | 9 | 2 |  |
| Constipation | 8 | 3 |  |
| Headache | 8 | 3 |  |
| Fatigue | 7 | 3 |  |
| Dizziness | 7 | 4 |  |
| Amenorrhea | 5 (out of 102 female) | 0 (out of 87 female) |  |
| Lactation | 4 (out of 102 female) | 0 (out of 87 female) |  |
| Dyspepsia | 2 | 5 |  |
| Increased appetite | 4 | 0 |  |
| Abdominal pain | 4 | 2 |  |
| Agitation | 1 | 3 |  |
| Abnormal accommodation | 1 | 3 |  |
| Sweating increased | 1 | 3 |  |
| Neurological events | 3 | 3 |  |
| **Thase (1996)** | **Sertraline (n=134)** | **Imipramine (n=136)** | **Placebo (n=140)** |
| Abnormal vision | 7 | 19 | 4 |
| Dry mouth | 35 | 100 | 24 |
| Flushing | 1 | 10 | 1 |
| Increased sweating | 16 | 39 | 8 |
| Micturition disorder | 1 | 15 | 1 |
| Palpitations | 6 | 12 | 3 |
| Postural hypotension | 0 | 7 | 0 |
| Dizziness | 19 | 38 | 22 |
| Headache | 55 | 53 | 64 |
| Paresthesia | 6 | 8 | 4 |
| Tremor | 14 | 33 | 1 |
| Anorexia | 16 | 15 | 4 |
| Constipation | 22 | 55 | 12 |
| Diarrhea | 28 | 10 | 14 |
| Dyspepsia | 17 | 17 | 9 |
| Increased appetite | 3 | 8 | 5 |
| Nausea | 37 | 35 | 28 |
| Vomiting | 6 | 10 | 2 |
| Agitation | 13 | 11 | 3 |
| Anxiety | 10 | 11 | 8 |
| Insomnia | 33 | 16 | 24 |
| Somnolence | 31 | 44 | 17 |
| Sexual dysfunction | 19 | 18 | 8 |
| Rash | 5 | 10 | 2 |
| **Vanelle (1997)** | **Fluoxetine (n=91)** | **Placebo (n=49)** |  |
| Neurological events | 22 | 17 |  |
| Gastrointestinal events | 11 | 8 |  |
| **Versiani (1997)** | **Moclobemide (n=108)** | **Imipramine (n=103)** | **Placebo (n=104)** |
| Dry mouth | 38 | 63 | 21 |
| Tremor | 10 | 26 | 7 |
| Constipation | 11 | 23 | 15 |
| Sweating | 5 | 18 | 1 |
| Blurred Vision | 5 | 10 | 4 |
| Sleepiness | 12 | 31 | 12 |
| Dizziness | 19 | 21 | 10 |
| Headache | 21 | 16 | 12 |
| Insomnia | 19 | 11 | 12 |
| Nausea | 19 | 15 | 10 |
| **Zanardi (2006)** | **Amisulpride (n=99)** | **Acetyl-L-Carnitine (n=105)** |  |
| Asthenia | 2 | 2 |  |
| Dyspepsia, dysphagia | 1 | 3 |  |
| Prolactin increased | 15 | 1 |  |
| Edema peripheral | 2 | 0 |  |
| Weight increased | 2 | 1 |  |
| Somnolence | 2 | 0 |  |
| Lactation | 6 | 0 |  |

CNS=central nervous system; ANS=autonomic nervous system
